# Racial and Ethnic Disparities in Survival Among People With Second Primary Cancer in the US

**DOI:** 10.1001/jamanetworkopen.2023.27429

**Published:** 2023-08-04

**Authors:** Hyuna Sung, Lauren Nisotel, Ephrem Sedeta, Farhad Islami, Ahmedin Jemal

**Affiliations:** 1 Surveillance & Health Equity Science, American Cancer Society, Atlanta, Georgia; 2 Department of Medicine, Brookdale University Hospital and Medical Center, Brooklyn, New York

## Abstract

**Importance:**

Comprehensive data for racial and ethnic disparities after second primary cancers (SPCs) are lacking despite the growing burden of SPCs.

**Objective:**

To quantify racial and ethnic disparities in survival among persons with SPCs.

**Design, Setting, and Participants:**

This population-based, retrospective cohort study used data from 18 Surveillance, Epidemiology, and End Results registries in the US for persons diagnosed with the most common SPCs at age 20 years or older from January 1, 2000, to December 31, 2013 (with follow-up through December 31, 2018). Data were analyzed between January and April 2023.

**Exposure:**

Race and ethnicity (Hispanic, non-Hispanic Asian or Pacific Islander, non-Hispanic Black, and non-Hispanic White).

**Main Outcomes and Measures:**

The main outcomes were 5-year relative survival and cause-specific survival. Cause-specific hazard ratios (HRs) were calculated for death from cancer or cardiovascular disease (CVD) in each racial and ethnic minority population compared with the White population overall and stratified by SPC type, with adjustment for sex, year and age at SPC diagnosis, and prior cancer type and stage (baseline model) and additionally for county attributes (household income, urbanicity), SPC characteristics (stage, subtype), and treatment.

**Results:**

Among 230 370 persons with SPCs (58.4% male), 4.5% were Asian or Pacific Islander, 9.6% were Black, 6.4% were Hispanic, and 79.5% were White. A total of 109 757 cancer-related deaths (47.6%) and 18 283 CVD-related deaths (7.9%) occurred during a median follow-up of 54 months (IQR, 12-93 months). In baseline models, compared with the White population, the risk of cancer-related death overall was higher in the Black (HR, 1.21; 95% CI, 1.18-1.23) and Hispanic (HR, 1.10; 95% CI, 1.07-1.13) populations but lower in the Asian or Pacific Islander population (HR, 0.93; 95% CI, 0.90-0.96). When stratified by 13 SPC types, the risk of cancer-related death was higher for 10 SPCs in the Black population, with the highest HR for uterine cancer (HR, 1.87; 95% CI, 1.63-2.15), and for 7 SPCs in the Hispanic population, most notably for melanoma (HR, 1.46; 95% CI, 1.21-1.76). For CVD-related death, the overall HR was higher in the Black population (HR, 1.41; 95% CI, 1.34-1.49), with elevated risks evident for 11 SPCs, but lower in the Asian or Pacific Islander (HR, 0.75; 95% CI, 0.69-0.81) and Hispanic (HR, 0.90; 95% CI, 0.84-0.96) populations than in the White population. After further adjustments for county attributes and SPC characteristics and treatment, HRs were reduced for cancer-related death and for CVD-related death and associations in the same direction remained.

**Conclusions and Relevance:**

In this cohort study of SPC survivors, the Black population had the highest risk of both death from cancer and death from CVD, and the Hispanic population had a higher risk of death from cancer than the White population. Attenuations in HRs after adjustment for potentially modifiable factors highlight opportunities to reduce survival disparities among persons with multiple primary cancers.

## Key Points

**Question:**

Are there racial and ethnic disparities in survival in the US population with second primary cancers?

**Findings:**

In this cohort study of 230 370 persons with second primary cancers, compared with the White population, the risk of cancer-related death was higher in the Black and Hispanic populations, whereas the risk of cardiovascular-related death was higher in the Black population but lower in the Asian or Pacific Islander and Hispanic populations.

**Meaning:**

The findings suggest that research priorities to address survival disparities in the growing population of survivors of multiple primary cancers are warranted.

## Teaser

This cohort study assesses racial and ethnic disparities in 5-year overall and cause-specific survival among adults with second primary cancer in the US.

## Introduction

Advances in early detection, treatment, and survivorship care for cancer have been associated with significant improvements in cancer survival over the past few decades in the US.^1^ Yet, substantial racial and ethnic disparities persist in cancer survival, reflecting systemic barriers to cancer screening, treatment, and survivorship care and differences in the prevalence of comorbidities.^2,3^ Most studies on racial and ethnic disparities in cancer survival, however, have compared survival among those with first primary cancer only or without distinction of tumor sequences.^1,2,3,4,5^ While evidence suggests that persons with multiple primary cancers have shorter survival compared with those with a single primary cancer,^6,7,8,9,10,11,12,13,14,15,16,17^ largely unknown are the magnitudes of racial and ethnic disparities in survival among persons with multiple primary cancers. One exception to this is a recent study of breast cancer survivors with second primary cancers (SPCs) that showed higher cancer and cardiovascular mortality among Black women and higher cancer mortality among Hispanic women compared with White women.^18^ The current study aimed to characterize racial and ethnic disparities in survival among persons diagnosed with 1 of the 13 most common SPCs, representing approximately 84% of all adult-onset SPCs.^19^

## Methods

### Study Population

This cohort study was based on deidentified, publicly available data and therefore was exempt from institutional review board approval or informed consent by the Morehouse School of Medicine. Persons diagnosed with SPCs at age 20 years or older from January 1, 2000, to December 31, 2013, were identified from 18 registries of the National Cancer Institute’s Surveillance, Epidemiology, and End Results (SEER) Program,^1^ covering 28% of the US population. This study followed the Strengthening the Reporting of Observational Studies in Epidemiology (STROBE) reporting guideline.

Second primary cancers were determined from tumor sequence numbers (ie, 02) and classified per the tenth *International Classification of Diseases for Oncology, Third Edition*.^20^ The top 10 most diagnosed SPCs for each sex were included based on a prior study (13 SPC types).^19^ In SEER, race and ethnicity are collected via medical records, which are either self-reported or inferred from a practitioner and classified into 5 categories^21^: Hispanic, non-Hispanic American Indian or Alaska Native, non-Hispanic Asian or Pacific Islander, non-Hispanic Black, and non-Hispanic White. American Indian or Alaska Native individuals were excluded due to a small number of SPC cases that precluded robust analysis, as were persons with unknown race and ethnicity, those identified with only autopsy or death certificate records, and those with incomplete survival data and follow-up information (eTable 1 in Supplement 1 gives details). Person-years at risk were calculated from the date of SPC diagnosis to the date of death, last living contact, or study end date (December 31, 2018), whichever came first.

### Outcomes

Two outcomes of interest were 5-year relative survival and cause-specific survival. For cause-specific survival, cause-of-death data from death certificates^22^ were classified to define 2 end points: death from any cancer and death from cardiovascular disease (CVD) (eTable 2 in Supplement 1), the top 2 most common causes of death among cancer survivors.^23,24^

### Statistical Analysis

All-stage and stage-specific, 5-year, age-standardized relative survival rates were calculated overall and for each SPC by race and ethnicity as a ratio of the observed survival from all causes of death for the patient cohort to the expected survival, as estimated by life tables for each population.^4,25^ To quantify differences in cause-specific survival across race and ethnicity, adjusted for differences in sociodemographic and clinical characteristics, hazard ratios (HRs) and 95% CIs were calculated using Cox proportional hazards regression models comparing the risk of death from cancer or CVD in Asian or Pacific Islander, Black, or Hispanic populations with that in the White population. Baseline models (model 1) were adjusted for sex, prior cancer type (eTable 3 in Supplement 1), prior cancer stage (localized, regional, distant, unknown or unstaged, or blank [cases with certain site and/or year combinations that were not covered by SEER historic stage A are coded as blank]^26^), and year (2000-2004, 2005-2009, and 2010-2013) and age (5-year interval) at SPC diagnosis. Model 2 was additionally adjusted for county-level annual household income (<$35 000-$59 000, $60 000-$74 999,≥$75 000, or unknown) and urbanicity (large metropolitan [≥1 million population], small metropolitan [<1 million population], nonmetropolitan, or unknown) based on Rural-Urban Continuum Codes developed by the US Department of Agriculture.^27^ Model 3 was further adjusted for SPC clinical characteristics (type [only for a combined analysis of all SPCs], stage, and subtype [eTable 4 in Supplement 1 gives details]) and receipt of treatment (surgery, radiotherapy, or chemotherapy). The Cox proportional hazards regression assumption was tested based on the Schoenfeld residuals plot. Variables that violated the assumptions (prior cancer type, SPC type, chemotherapy receipt, or radiotherapy receipt) were treated as strata to allow the baseline hazards to differ. Heterogeneities in the associations by select variables were examined by stratified analysis with interactions between race and ethnicity and the variable of interest tested using a likelihood ratio test. Sensitivity analyses quantified survival differences (1) in the presence of competing risks using the Fine-Gray subdistribution hazard model^28^ and (2) in cause-specific models using age as the time scale.^29^ Analyses were performed using SEER*Stat, version 8.3.6 (National Cancer Institute)^3^ and SAS, version 9.4 (SAS Institute Inc) between January and April 2023. Two-sided *P* < .05 was considered statistically significant.

## Results

Of 230 370 persons with SPCs (41.6% female; 58.4% male), 4.5% were Asian or Pacific Islander, 9.6% were Black, 6.4% were Hispanic, and 79.5% were White. Sociodemographic and clinical characteristics varied by race and ethnicity (Table 1). Asian or Pacific Islander, Black, and Hispanic populations were more likely than the White population to be younger at SPC diagnosis and to live in large metropolitan areas, while the Black population was most likely to live in counties with the lowest household income. Asian or Pacific Islander, Black, and Hispanic populations were less likely than the White population to be diagnosed with localized-stage SPCs (Asian or Pacific Islander, 50.6%; Black, 48.1%; and Hispanic, 51.2% vs White, 55.0%), and the Black population was least likely to undergo surgery (53.5% vs Asian or Pacific Islander, 63.0%; Hispanic, 61.7%; White, 62.4%). Similar patterns were observed across SPC types, with unfavorable (localized) stage distribution most notable for breast cancer, uterine cancer, and melanoma of the skin between Black and White populations (breast: 57.8% vs 70.1%; uterine: 53.1% vs 61.8%; melanoma: 67.3% vs 85.3%) (eFigure 1 and eTable 5 in Supplement 1). Distribution in SPC subtypes also varied by race and ethnicity (eFigure 1 in Supplement 1). Notably, Black and Hispanic women more frequently presented with aggressive forms of cancers compared with White women (eg, hormone receptor–negative breast cancer: Black, 29.6%; Hispanic, 22.2%; White, 15.9%; nonendometrioid of corpus uteri: Black, 37.9%; Hispanic, 27.6%; White, 21.7%).

**Table 1.** Vital Status and Sociodemographic and Clinical Characteristics by Race and Ethnicity Among Persons Diagnosed at Age 20 Years or Older With SPCs From 2000 to 2013 in 18 SEER Registries

Characteristic	Race and ethnicity^a^				
	Asian or Pacific Islander (n = 10 294)	Black (n = 22 077)	Hispanic (n = 14 840)	White (n = 183 159)	Total (N = 230 370)
Follow-up, median (IQR), mo	60 (12-96)	38 (9-86)	55 (12-92)	55 (12-94)	54 (12-93)
Vital status and cause of death					
Alive	4199 (40.8)	6600 (29.9)	5807 (39.1)	63 617 (34.7)	80 223 (34.8)
Cause of death					
Cancer	4794 (46.6)	11 811 (53.5)	7041 (47.4)	86 111 (47.0)	109 757 (47.6)
Cardiovascular disease	594 (5.8)	1737 (7.9)	853 (5.7)	15 099 (8.2)	18 283 (7.9)
Other	707 (6.9)	1929 (8.7)	1139 (7.7)	18 332 (10.0)	22 107 (9.6)
Age at SPC diagnosis, y					
20-49	870 (8.5)	1526 (6.9)	1609 (10.8)	8114 (4.4)	12 119 (5.3)
50-64	2781 (27.0)	7055 (32.0)	4143 (27.9)	43 123 (23.5)	57 102 (24.8)
65-74	2957 (28.7)	7532 (34.1)	4481 (30.2)	59 074 (32.3)	74 044 (32.1)
≥75	3686 (35.8)	5964 (27.0)	4607 (31.0)	72 848 (39.8)	87 105 (37.8)
Year at SPC diagnosis					
2000-2004	1327 (12.9)	3319 (15.0)	1923 (13.0)	28 200 (15.4)	34 769 (15.1)
2005-2009	3974 (38.6)	8728 (39.5)	5610 (37.8)	72 576 (39.6)	90 888 (39.5)
2010-2013	4993 (48.5)	10 030 (45.4)	7307 (49.2)	82 383 (45.0)	104 713 (45.5)
Sex					
Female	5047 (49.0)	9086 (41.2)	7070 (47.6)	74 652 (40.8)	95 855 (41.6)
Male	5247 (51.0)	12 991 (58.8)	7770 (52.4)	108 507 (59.2)	134 515 (58.4)
Annual household income, $					
<35 000-59 999	594 (5.8)	9675 (43.8)	3410 (23.0)	57 702 (31.5)	71 381 (31.0)
60 000-74 999	4157 (40.4)	8590 (38.9)	7811 (52.6)	69 232 (37.8)	89 790 (39.0)
≥75 000	5543 (53.8)	3810 (17.3)	3609 (24.3)	56 203 (30.7)	69 165 (30.0)
Unknown	0	2 (<0.1)	10 (<0.1)	22 (<0.1)	34 (<0.1)
Urbanicity					
Large metropolitan	7184 (69.8)	14 982 (67.9)	10 255 (69.1)	106 398 (58.1)	138 819 (60.3)
Small metropolitan	2694 (26.2)	5189 (23.5)	3879 (26.1)	51 136 (27.9)	62 898 (27.3)
Nonmetropolitan	416 (4.0)	1904 (8.6)	696 (4.7)	25 603 (14.0)	28 619 (12.4)
Unknown	0	2 (<0.1)	10 (0.1)	22 (<0.1)	34 (<0.1)
SPC type					
Lung and bronchus	1958 (19.0)	5196 (23.5)	2242 (15.1)	38 670 (21.1)	48 066 (20.9)
Breast	1786 (17.3)	3300 (14.9)	2496 (16.8)	22 253 (12.1)	29 835 (13.0)
Colon and rectum	1560 (15.2)	3453 (15.6)	2135 (14.4)	21 275 (11.6)	28 423 (12.3)
Prostate	823 (8.0)	2702 (12.2)	1530 (10.3)	20 059 (11.0)	25 114 (10.9)
Urinary bladder	716 (7.0)	1314 (6.0)	1143 (7.7)	18 273 (10.0)	21 446 (9.3)
Non-Hodgkin lymphoma	679 (6.6)	1098 (5.0)	1218 (8.2)	13 353 (7.3)	16 348 (7.1)
Melanoma of the skin	92 (0.9)	104 (0.5)	334 (2.3)	15 901 (8.7)	16 431 (7.1)
Kidney and renal pelvis	443 (4.3)	1609 (7.3)	1091 (7.4)	9169 (5.0)	12 312 (5.3)
Oral cavity and pharynx	399 (3.9)	752 (3.4)	461 (3.1)	6611 (3.6)	8223 (3.6)
Pancreas	392 (3.8)	911 (4.1)	537 (3.6)	5864 (3.2)	7704 (3.3)
Stomach	566 (5.5)	771 (3.5)	581 (3.9)	3227 (1.8)	5145 (2.2)
Thyroid	511 (5.0)	379 (1.7)	645 (4.3)	4400 (2.4)	5935 (2.6)
Corpus uteri and uterus, not otherwise specified	369 (3.6)	488 (2.2)	427 (2.9)	4104 (2.2)	5388 (2.3)
Stage at SPC diagnosis					
Localized	5212 (50.6)	10 622 (48.1)	7591 (51.2)	100 725 (55.0)	124 150 (53.9)
Regional	2214 (21.5)	4635 (21.0)	3130 (21.1)	34 740 (19.0)	44 719 (19.4)
Distant	1980 (19.2)	4945 (22.4)	2772 (18.7)	33 308 (18.2)	43 005 (18.7)
Unknown or unstaged	633 (6.1)	1225 (5.5)	994 (6.7)	8985 (4.9)	11 837 (5.1)
Blank^b^	255 (2.5)	650 (2.9)	353 (2.4)	5401 (2.9)	6659 (2.9)
Surgery for SPC					
Performed	6487 (63.0)	11 807 (53.5)	9149 (61.7)	114 308 (62.4)	141 751 (61.5)
Not performed	3661 (35.6)	10 062 (45.6)	5560 (37.5)	67 207 (36.7)	86 490 (37.5)
Unknown	146 (1.4)	208 (0.9)	131 (0.9)	1644 (0.9)	2129 (0.9)
Radiotherapy for SPC					
Yes	2289 (22.2)	5103 (23.1)	2914 (19.6)	38 485 (21.0)	48 791 (21.2)
None or unknown	8005 (77.8)	16 974 (76.9)	11 926 (80.4)	144 674 (79.0)	181 579 (78.8)
Chemotherapy for SPC					
Yes	2683 (26.1)	5870 (26.6)	3670 (24.7)	40 783 (22.3)	53 006 (23.0)
No or unknown	7611 (73.9)	16 207 (73.4)	11 170 (75.3)	142 376 (77.7)	177 364 (77.0)
Time between prior cancer diagnosis and SPC diagnosis, y					
0-4.9	6910 (67.1)	14 707 (66.6)	10 000 (67.4)	122 511 (66.9)	154 128 (66.9)
5-9.9	2824 (27.4)	6107 (27.7)	3955 (26.7)	50 067 (27.3)	62 953 (27.3)
≥10	560 (5.4)	1263 (5.7)	885 (6.0)	10 581 (5.8)	13 289 (5.8)
Prior cancer type					
Genital system	2271 (22.1)	6829 (30.9)	3599 (24.3)	42 474 (23.2)	55 173 (23.9)
Breast	2162 (21.0)	3975 (18.0)	2907 (19.6)	27 861 (15.2)	36 905 (16.0)
Digestive system	2195 (21.3)	3738 (16.9)	2566 (17.3)	24 000 (13.1)	32 499 (14.1)
Urinary system	1041 (10.1)	2244 (10.2)	1905 (12.8)	26 117 (14.3)	31 307 (13.6)
Respiratory system	762 (7.4)	2102 (9.5)	869 (5.9)	16 105 (8.8)	19 838 (8.6)
Lymphoma, myeloma, or leukemia	698 (6.8)	1490 (6.7)	1290 (8.7)	14 872 (8.1)	18 350 (8.0)
Skin or eye	115 (1.1)	137 (0.6)	373 (2.5)	16 237 (8.9)	16 862 (7.3)
Oral cavity and pharynx	411 (4.0)	698 (3.2)	426 (2.9)	6606 (3.6)	8141 (3.5)
Endocrine system	352 (3.4)	299 (1.4)	442 (3.0)	3446 (1.9)	4539 (2.0)
Bones and joints or soft tissue including heart	53 (0.5)	115 (0.5)	130 (0.9)	1037 (0.6)	1335 (0.6)
Brain and other nervous system	23 (0.2)	34 (0.2)	35 (0.2)	437 (0.2)	529 (0.2)
Other	211 (2.0)	416 (1.9)	298 (2.0)	3967 (2.2)	4892 (2.1)
Age at prior cancer diagnosis, y					
20-49	1354 (13.2)	2523 (11.4)	2372 (16.0)	14 087 (7.7)	20 336 (8.8)
50-64	3319 (32.2)	8790 (39.8)	4965 (33.5)	57 914 (31.6)	74 988 (32.6)
65-74	3102 (30.1)	7157 (32.4)	4499 (30.3)	62 025 (33.9)	76 783 (33.3)
≥75	2519 (24.5)	3607 (16.3)	3004 (20.2)	49 133 (26.8)	58 263 (25.3)
Year at prior cancer diagnosis					
2000-2004	4942 (48.0)	11 243 (50.9)	6956 (46.9)	95 603 (52.2)	118 744 (51.5)
2005-2009	3993 (38.8)	8215 (37.2)	5794 (39.0)	66 828 (36.5)	84 830 (36.8)
2010-2013	1359 (13.2)	2619 (11.9)	2090 (14.1)	20 728 (11.3)	26 796 (11.6)
Stage at prior cancer diagnosis					
Localized	5870 (57.0)	13 113 (59.4)	8426 (56.8)	116 647 (63.7)	144 056 (62.5)
Regional	2504 (24.3)	4785 (21.7)	3471 (23.4)	34 683 (18.9)	45 443 (19.7)
Distant	777 (7.5)	1885 (8.5)	1244 (8.4)	11 968 (6.5)	15 874 (6.9)
Unknown or unstaged	329 (3.2)	804 (3.6)	577 (3.9)	5009 (2.7)	6719 (2.9)
Blank^b^	814 (7.9)	1490 (6.7)	1122 (7.6)	14 852 (8.1)	18 278 (7.9)

Abbreviations: SEER, Surveillance, Epidemiology, and End Results; SPC, second primary cancer.

^a^ Data are presented as number (percentage) of persons unless otherwise indicated. All race categories are exclusive of Hispanic ethnicity.

^b^ Cases with certain site and/or year combinations that were not covered by SEER historic stage A were coded as blank, including 3716 oral cancer cases for certain subsites from 2004 to 2013 and 2943 chronic lymphocytic leukemia cases.^26^

**Figure 1.**
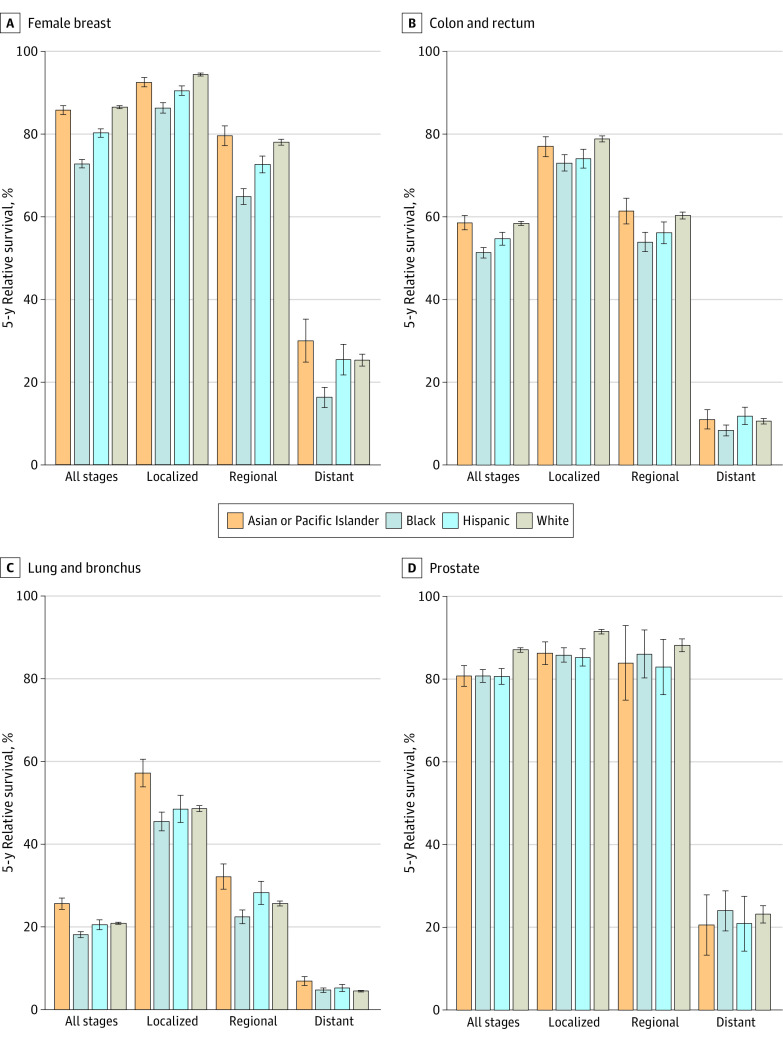
Five-Year, Age-Standardized Relative Survival for the 4 Most Common Second Primary Cancers by Race and Ethnicity All race categories are exclusive of Hispanic ethnicity. Whiskers indicate 95% CIs. Results for remaining second primary cancers are shown in eFigure 2 in Supplement 1.

During a median follow-up of 54 months, 109 757 cancer-related deaths (47.6%) and 18 283 CVD-related deaths (7.9%) occurred among persons with SPCs. Figure 1 shows the 5-year relative survival for the 4 most common SPCs by race and ethnicity (eFigure 2 in Supplement 1 gives results for all SPCs combined and the remaining SPCs). For all SPCs and stages combined, relative survival was highest in the White population (60.6%; 95% CI, 60.5%-60.8%), closely followed by the Asian or Pacific Islander (59.3%; 95% CI, 58.7%-60.0%) and Hispanic (58.1%; 95% CI, 57.5%-58.6%) populations, and was lowest in the Black population (50.6%; 95% CI, 50.1%-51.1%). The White population had the highest survival of all populations for 8 of 13 SPCs, with the difference greatest for melanoma (85.5% vs Asian or Pacific Islander, 68.2%; Black, 58.5%; Hispanic, 71.2%) and non-Hodgkin lymphoma (63.5% vs Asian or Pacific Islander, 55.7%; Black, 56.6%; Hispanic, 54.5%). The Asian or Pacific Islander population had the highest survival for cancers of the colorectum, pancreas, stomach, and lung but the lowest for thyroid cancer. The Black population had the lowest survival for 10 of 13 SPCs, with the difference from the White population most pronounced for uterine cancer (51.6% vs 73.4%) and melanoma (58.5% vs 85.5%). Compared with the White population, relative survival was also lower for 10 SPCs in the Hispanic population and 7 SPCs in the Asian or Pacific Islander population.

Table 2 shows the overall adjusted HRs for the associations of race and ethnicity with risk of cancer-related death or CVD-related death among persons with SPCs. In baseline models (model 1), compared with the White population, the risk of cancer-related death was higher in the Black (HR, 1.21; 95% CI, 1.18-1.23) and Hispanic (HR, 1.10; 95% CI, 1.07-1.13) populations but lower in the Asian or Pacific Islander population (HR, 0.93; 95% CI, 0.90-0.96). Similarly, the risk of CVD-related death was higher in the Black population (HR, 1.41; 95% CI, 1.34-1.49), with elevated risks evident for 11 SPCs, and lower in the Asian or Pacific Islander population (HR, 0.75; 95% CI, 0.69-0.81) than in the White population; the risk was reversed in the Hispanic population (HR, 0.90; 95% CI, 0.84-0.96). After additional adjustments for county attributes (model 2) and SPC characteristics and treatment (model 3), HRs were reduced for cancer-related death in the Black and Hispanic populations and CVD-related death in the Black population and associations in the same directions remained in these groups.

**Table 2.** Association of Race and Ethnicity With Risk of Death From Cancer or CVD Among Persons Diagnosed at Age 20 Years or Older With SPCs From 2000 to 2013 in 18 SEER Registries

Cause of death, race and ethnicity^a^	Deaths, No./total No. (%)	HR (95% CI)			HR reduction from model 1 to model 3, %^e^
		Model 1^b^	Model 2^c^	Model 3^d^	
Cancer					
Asian or Pacific Islander	5500/10 294 (53.4)	0.93 (0.90-0.96)	0.98 (0.95-1.01)	0.90 (0.86-0.93)	NC
Black	10 266/22 077 (46.5)	1.21 (1.18-1.23)	1.19 (1.17-1.21)	1.08 (1.05-1.11)	61.0
Hispanic	7799/14 840 (52.6)	1.10 (1.07-1.13)	1.11 (1.09-1.14)	1.04 (1.00-1.07)	63.4
White	97 048/183 159 (53.0)	1 [Reference]	1 [Reference]	1 [Reference]	NC
CVD					
Asian or Pacific Islander	594/10 294 (5.8)	0.75 (0.69-0.81)	0.78 (0.72-0.85)	0.78 (0.69-0.87)	NC
Black	1737/22 077 (7.9)	1.41 (1.34-1.49)	1.39 (1.32-1.46)	1.30 (1.21-1.4)	26.7
Hispanic	853/14 840 (5.7)	0.90 (0.84-0.96)	0.90 (0.84-0.97)	0.89 (0.8-0.98)	NC
White	15 099/183 159 (8.2)	1 [Reference]	1 [Reference]	1 [Reference]	NC

Abbreviations: CVD, cardiovascular diease; HR, hazard ratio; NC, not calculated; SEER, Surveillance, Epidemiology, and End Results; SPC, second primary cancer.

^a^ All race categories are exclusive of Hispanic ethnicity.

^b^ Adjusted for sex (female, male), prior cancer type or system (eTable 3 in Supplement 1), prior cancer stage (localized, regional, distant, unknown or unstaged, or blank), and year (2000-2004, 2005-2009, or 2010-2013) and age (5-year interval) at SPC diagnosis.

^c^ Adjusted for the variables in model 1 and county-level annual household income (<$35 000-$59 000, $60 000-$74 999, ≥$75 000, or unknown) and county-level urbanity (large metropolitan, small metropolitan, nonmetropolitan, or unknown).

^d^ Adjusted for the variables in model 2 and SPC type, stage, subtype (eTable 4 in Supplement 1), and treatment (including receipt of surgery, radiotherapy, or chemotherapy for SPC).

^e^ Calculated using the formula only when the HRs in models 1 and 3 were higher than 1.00: [model 1 HR − model 3 HR] / [model 1 HR] × 100.

**Figure 2.**
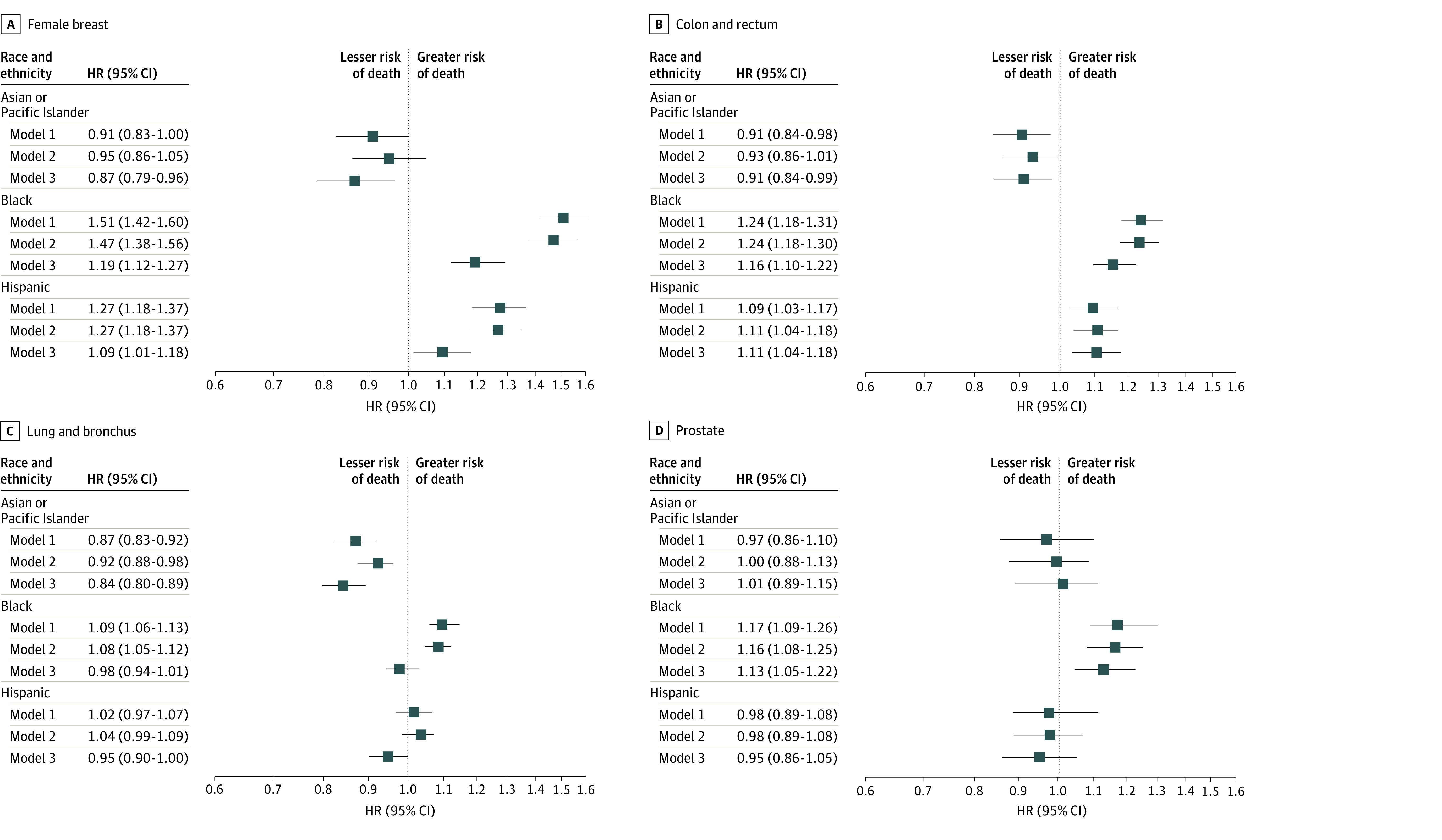
Association of Race and Ethnicity With Risk of Death From Cancer Among Persons With Second Primary Cancers (SPCs) All race categories are exclusive of Hispanic ethnicity. In Cox proportional hazards regression, sequentially adjusted covariates included sex (female, male), prior cancer type or system (eTable 3 in Supplement 1), prior cancer stage (localized, regional, distant, unknown or unstaged, or blank), and year (2000-2004, 2005-2009, or 2010-2013) and age (5-year interval) at SPC diagnosis for model 1; county-level annual household income (<$35 000-$59 000, $60 000-$74 999, ≥$75 000, or unknown) and county-level urbanity (large metropolitan, small metropolitan, nonmetropolitan, or unknown) for model 2; and SPC type, stage, subtype (eTable 4 in Supplement 1), and treatment (including receipt of surgery, radiotherapy, or chemotherapy for SPC) for model 3. The White population was the reference. Squares represent hazard ratios (HRs), with horizontal lines representing 95% CIs. Point estimates and 95% CIs for the remaining SPCs along with percentage reductions in HRs between models 1 and 3 are given in eFigure 3 and eTable 6 in Supplement 1.

**Figure 3.**
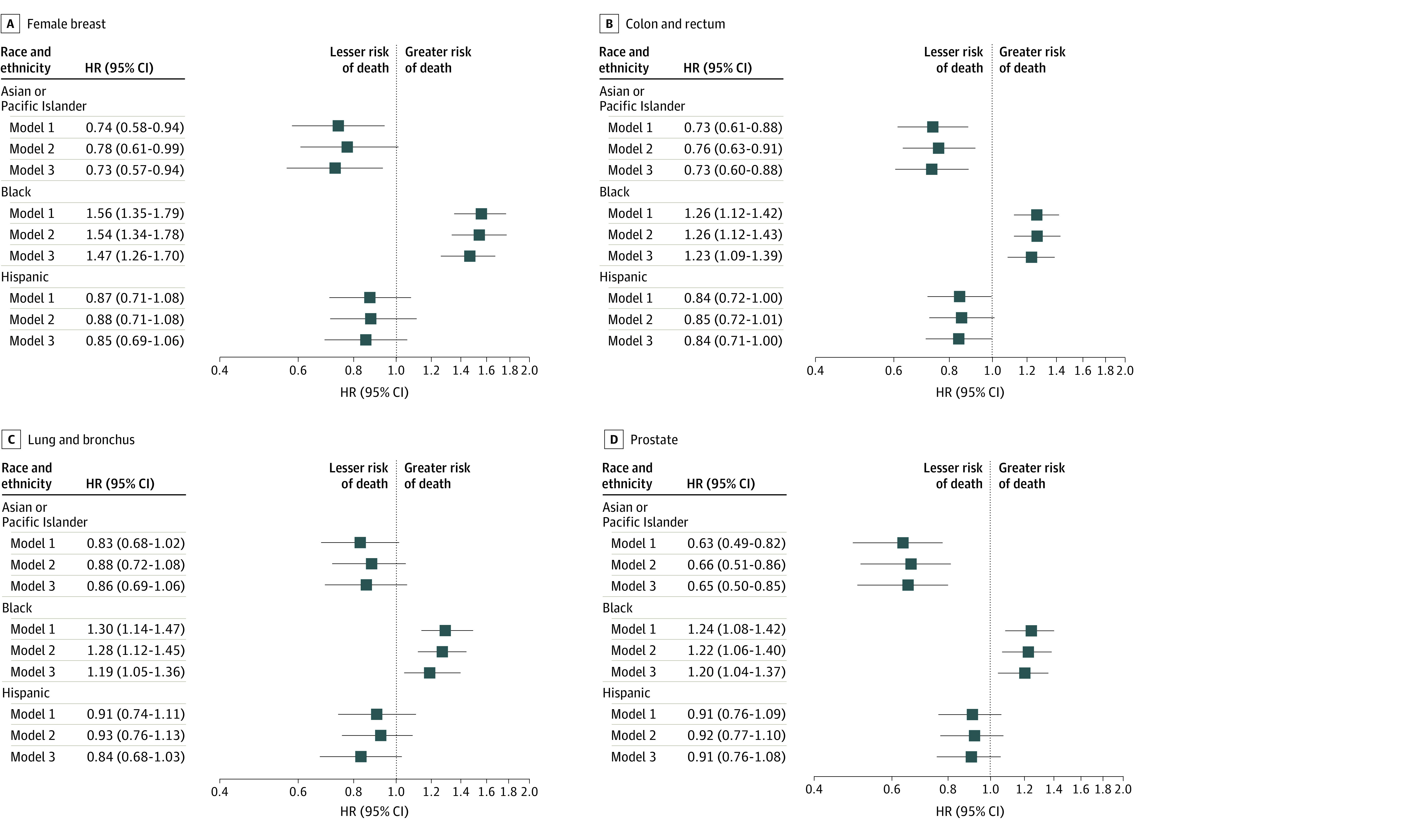
Association of Race and Ethnicity With Risk of Death From Cardiovascular Disease Among Persons With Second Primary Cancers (SPCs) All race categories are exclusive of Hispanic ethnicity. In the Cox proportional hazards regression, sequentially adjusted covariates included sex (female, male), prior cancer type or system (eTable 3 in Supplement 1), prior cancer stage (localized, regional, distant, unknown or unstaged, or blank), and year (2000-2004, 2005-2009, or 2010-2013) and age (5-year interval) at SPC diagnosis for model 1; county-level annual household income (<$35 000-$59 000, $60 000-$74 999, ≥$75 000, or unknown) and county-level urbanity (large metropolitan, small metropolitan, nonmetropolitan, or unknown) for model 2; and SPC type, stage, subtype (eTable 4 in Supplement 1), and treatment (including receipt of surgery, radiotherapy, or chemotherapy for SPC) for model 3. The White population was the reference. Squares represent hazard ratios (HRs), with horizontal lines representing 95% CIs. Point estimates and 95% CIs for the remaining SPCs along with percentage reductions in HRs between models 1 and 3 are given in eFigure 4 and eTable 7 in Supplement 1.

Figure 2 and Figure 3 show HRs stratified by the 4 most common SPCs for risk of cancer-related death and CVD-related death, respectively. The corresponding results for the remaining SPCs are presented in eFigures 3 and 4 in Supplement 1, respectively. In model 1, the Black population had a significantly higher risk for cancer-related death than the White population for 10 of 13 SPCs, with the greatest HRs for uterine cancer (HR, 1.87; 95% CI, 1.63-2.15), melanoma (HR, 1.58; 95% CI, 1.17-2.12), and breast cancer (HR, 1.51; 95% CI, 1.42-1.60) (Figure 2 and eFigure 3 in Supplement 1). Similarly, the Hispanic population had a significantly higher risk for cancer-related death than the White population for 7 SPCs, most notably for melanoma (HR, 1.46; 95% CI, 1.21-1.76), uterine cancer (HR, 1.34; 95% CI, 1.14-1.59), and breast cancer (HR, 1.27; 95% CI, 1.18-1.37), but they had similar risks for the remaining SPCs. Despite the overall lower risk of cancer-related death in the Asian or Pacific Islander population, this population had a significantly higher risk of cancer-related death than the White population for melanoma (HR, 1.93; 95% CI, 1.42-2.62), thyroid cancer (HR, 1.26; 95% CI, 1.04-1.54), and non-Hodgkin lymphoma (HR, 1.13; 95% CI, 1.01-1.27). After full adjustment for all covariables, HRs were reduced in the Black and Hispanic populations, with the greatest percentage reductions for lung (in the Black population) and breast and uterine cancers (in the Black and Hispanic populations) and associations in the same directions remained (eTable 6 in Supplement 1). Notably, HRs were reduced to nonsignificant levels in the fully adjusted models for lung cancer, melanoma, and pancreatic cancer in the Black population; uterine cancer in the Hispanic population; and thyroid cancer and non-Hodgkin lymphoma in the Asian or Pacific Islander population.

For the risk of CVD-related death, based on model 1, the Black population had a significantly higher risk than the White population for all SPCs except uterine cancer and melanoma, with the highest HRs for pancreatic (HR, 1.80; 95% CI, 1.17-2.75), thyroid (HR, 1.70; 95% CI, 1.12-2.57), and kidney (HR, 1.63; 95% CI, 1.38-1.93) cancers (eFigure 4 in Supplement 1). In the Asian or Pacific Islander and Hispanic populations, HRs were lower than in the White population for most SPCs, albeit statistically significant only for thyroid, prostate, colorectal, urinary bladder, and breast cancers in the Asian or Pacific Islander population (range was from HR, 0.31; 95% CI, 0.14-0.71 for thyroid cancer to HR, 0.74; 95% CI, 0.58-0.94 for breast cancer) and for colorectal cancer in the Hispanic population (HR, 0.84; 95% CI, 0.72-1.00) (Figure 3 and eFigure 4 in Supplement 1). After full adjustment, HRs were reduced for cancer-related death and CVD-related death and associations in the same directions remained (eTable 7 in Supplement 1).

Associations for all SPCs combined were generally consistent across strata of age, sex, stage, SPC subtypes, and county attributes (eFigure 5 and eTable 8 in Supplement 1). However, the increased risk of cancer-related death in the Black and Hispanic populations was greater for those diagnosed with localized or regional SPCs and at younger ages (20-64 years vs ≥65 years) and among females in only the Black population, whereas the increased risk of CVD-related death in the Black population was highest for those diagnosed with distant-stage diseases and at younger ages. Survival advantages in the Asian or Pacific Islander population tended to be higher in counties with lower household income, albeit there was not a significant difference. In competing risk models, the direction and statistical significance of the associations were the same as the cause-specific models, although risk was lower especially in the Black population, likely reflecting a greater impact from mortality of competing risks (eTable 9 in Supplement 1). Models using age as a time scale generated comparable results (eTable 10 in Supplement 1).

## Discussion

In this diverse, contemporary, population-based cohort study of 230 370 persons with SPCs, the Black and Hispanic populations compared with the White population had a higher risk of cancer-related death, with the greatest disparities commonly observed for persons with SPCs of the breast and corpus uteri and melanoma. The Black population also experienced the greatest risk of death from CVD of all populations overall and across most SPCs. Over 20% of newly diagnosed cancers in the US occur among persons with a cancer history,^30,31^ and the proportion is expected to increase,^30^ highlighting a critical need to better understand outcomes in persons with multiple primary cancers. Complementing the currently expanding knowledge on SPC risk and care,^6,7,8,9,10,11,12,13,14,15,16,17^ the findings of racial and ethnic disparities in SPC survival may inform the development and equitable implementation of survivorship and treatment guidelines for the growing population of persons with multiple primary cancers.

Racial and ethnic disparities in SPC survival were largely consistent with those reported for first primary cancers, with the Black population experiencing the highest cancer mortality for most SPCs.^3,4,5^ Lower SPC survival in the Hispanic population compared with the White population was attributable to those with SPCs of the breast, colorectum, and corpus uteri; non-Hodgkin lymphoma; and melanoma. This finding is somewhat different from previous studies of first primary cancers that showed comparable survival between Hispanic and non-Hispanic White populations for multiple major cancers.^3,4,5^ Unfavorable stage distributions at SPC diagnosis were evident in the Black and Hispanic populations, particularly for breast cancer, uterine cancer, and melanoma, and in the Asian or Pacific Islander population for thyroid cancer and melanoma. Together with previous observations on lower use of surveillance tests for SPCs reported among Black or Hispanic cancer survivors than among their White counterparts,^32,33,34^ the findings suggest that reducing disparities in stage distribution is an important pathway toward equitable outcomes.

Future research is needed to address barriers to obtaining surveillance tests differentially experienced by racial and ethnic groups; these barriers may include greater medical and nonmedical financial hardship and lack of physicians’ recommendations and health literacy among cancer survivors.^35,36,37,38^ Of note, greater disparities in cancer-related death found among those with localized or regional-stage SPCs may reflect various highly successful treatment options and better survival among those who received such care in contrast to distant-stage disease with generally few treatment options and poor prognosis. Nevertheless, substantial reductions in HRs for cancer-related death after adjustment for SPC characteristics and treatment receipt suggest opportunities to mitigate the survival disparities via equitable access to early detection and stage-appropriate, guideline-concordant care.

In addition to unfavorable stage distribution, varying subtype distributions by race and ethnicity also likely contributed to disparities in SPC survival. Closely resembling literature of first primary cancers,^3,39,40,41,42,43,44^ Black women were most likely to present with aggressive forms of second breast and uterine cancers, whereas the relatively less aggressive type of lung cancer, non–small cell adenocarcinoma, was most common in the Asian or Pacific Islander population. Albeit less prominent than in Black women, unfavorable subtype distributions were also found among Hispanic women with breast and uterine cancers in contrast to previous studies of first primary cancers,^39,40^ which may, in part, explain the aforementioned discrepancies in survival patterns in the Hispanic population.

The patterns in racial and ethnic disparities in CVD-related death were consistent with previous studies of populations with or without cancer^3,45^ demonstrating that detrimental effects of disparities in cardiovascular health in the general population may extend to cancer survivors.^46,47^ Reasons for the highest risk of CVD-related death found in the Black population with SPCs were likely multifactorial and may have largely reflected a disproportionately higher burden of CVD and risk factors (eg, hypertension, diabetes, metabolic syndrome, obesity, and kidney disease) reported in the Black population in various settings.^48,49,50,51,52^ The unfavorable cardiovascular health profile may have been exacerbated among cancer survivors given the substantial overlap between cancer and CVD risk factors.^53,54^ A greater disparity among individuals younger than 65 years may have reflected larger disparities in access to health care partly attributable to aggravated disparities in health insurance coverage.^55,56^ Reasons for the worsening disparities among patients with advanced-stage cancer are unclear but may be related to observations of a higher prevalence of cardiotoxic effects after nonsurgical therapies among Black vs White persons.^57,58,59,60,61,62,63^

The lower risk of CVD-related death in the Asian or Pacific Islander and Hispanic populations than in the White population remained similar after adjustment for differences in county attributes and SPC characteristics and treatment, suggesting an association with other unmeasured factors. The tendency of more pronounced survival advantage in the Asian or Pacific Islander population in the counties with lower household income may be associated with a prior notion that adverse health effects of living in resource-limited areas seem somewhat alleviated in low-income neighborhoods and areas with large immigrant populations (often referred to as the immigrant paradox),^64,65^ possibly because of mechanisms of increased social cohesion, family support, lower smoking prevalence, and better diet quality.^66,67^ However, not all ethnic groups benefited equally from potential resilience factors given the widely differing profiles of social conditions and cardiovascular outcomes reported in subpopulations of the Asian or Pacific Islander and Hispanic populations.^68,69,70,71^ Future studies with disaggregated data are needed to better understand disparities, needs, and opportunities unique to specific ethnic groups in these understudied populations.

Leveraging large population-based data, this study provided an extent of racial and ethnic disparities in survival among persons with SPCs overall and across a comprehensive list of SPC types. Considering substantial racial and ethnic disparities in SPC survival, barriers to providing high-quality treatment and survivorship care should be identified across diverse populations of patients with multiple primary cancers and different social and clinical needs. However, quality care metrics are currently lacking largely due to a lack of well-established treatment and survivorship guidelines tailored for the complex and evolving needs of persons with multiple primary cancers.^72^ Development of these guidelines should consider various challenges faced by persons with multiple primary cancers,^73^ such as balancing the benefits and harms of intensive cancer screening, limited treatment options concerning drug resistance and contraindication,^13,74^ the possibility of underlying factors associated with cancer, multiple chronic morbidities,^75^ the complexity in navigating health care systems (multiple referrals and care transitions between care settings and health care practitioners), and exacerbated financial hardship.^76^ Issues of financial hardship may be particularly relevant to the observed racial and ethnic disparities in SPC survival, as prior cancer-related disruptions and employment consequences are disproportionately experienced by racial and ethnic minority groups,^77^ with the Black and Hispanic populations being significantly more likely to be denied health insurance due to a prior cancer diagnosis compared with the non-Hispanic White population.^77,78^

### Limitations

This study has several limitations. First, causes of death ascertained from death certificate information are subject to misclassification,^79,80^ and the accuracy of cause of death among persons with multiple primary cancers remains to be determined. Second, misclassification of race and ethnicity was also likely but expected to be minimal^81^ given that validated algorithms are currently used in SEER registries to improve the classification.^82^

Third, limitations inherent in cancer registry data prohibited detailed analysis of specific patient, clinical, and health care system factors that likely affected the observed disparities. In particular, data for comorbid conditions and the receipt of guideline-concordant care were unavailable in the SEER registries, and their association with racial and ethnic disparities could not be considered. Additionally, county-level household income and urbanicity are crude measures to adequately capture individuals’ socioeconomic profile and neighborhood characteristics that may affect SPC control and care.^83,84^ Future studies in diverse groups with data for social and geospatial determinants of health at multiple levels (eg, social deprivation, residential segregation, and transportation), various exposures (eg, smoking, obesity), comorbid conditions, genetic and molecular tumor characteristics,^85,86^ access to and affordability of health care, and adherence to surveillance and treatment for SPCs and CVDs^87^ are needed to measure the extent of racial and ethnic disparities in SPC survival that are avoidable and inform interventions to narrow the gap.

## Conclusions

In this cohort study of a US population with SPCs, the Black population had a higher risk of death from both cancer and CVD compared with the White population, whereas the Hispanic population had a higher risk of death from cancer. These findings suggest that research priorities to address survival disparities in the growing population of survivors of multiple primary cancers are warranted.

## Notes

Supplement 1. **eTable 1.** Participant Selection Process SEER Research Plus Data, 18 Registries (Excl AK), Nov 2020 Sub (2000-2018) **eTable 2.** Definition of Cardiovascular Diseases and International Classification of Diseases (ICD)-10 Codes **eTable 3.** Classification of Prior Cancer Type **eTable 4.** Definition of Subtypes for Second Primary Cancers of the Female Breast, Lung and Bronchus, Corpus Uteri, and Non-Hodgkin Lymphoma **eTable 5.** Distribution of Treatment Receipt for Second Primary Cancer by Race and Ethnicity **eTable 6.** Associations of Race and Ethnicity With the Risk of Cancer Death Among Persons With Second Primary Cancers (SPCs) (Reference: White) **eTable 7.** Associations of Race and Ethnicity With the Risk of Cardiovascular Death Among Persons With Second Primary Cancers (SPCs) (Reference: White) **eTable 8.** Association of Race and Ethnicity With the Risk of Cancer or Cardiovascular Death According to Second Primary Cancer (SPC) Subtype (Reference: White) **eTable 9.** Association of Race and Ethnicity With the Risk of Cancer or Cardiovascular Death in the Fine-Gray Model Among Persons With Second Primary Cancer (SPCs) **eTable 10.** Association of Race and Ethnicity With the Risk of Cancer or Cardiovascular Death in Cox Proportional Hazards Models Using Age as a Time-Scale Among Persons With Second Primary Cancers (SPCs) **eFigure 1.** Distribution of (A) County-Level Household Income, (B) County-Level Urbanicity, (C) Stage at Second Primary Cancer Diagnosis, and (D) Second Primary Cancer Subtype by Race and Ethnicity According to Second Primary Cancer Type **eFigure 2.** Five-Year, Age-Standardized Relative Survival of Second Primary Cancers by Race and Ethnicity, Overall and by Stage **eFigure 3.** Association of Race and Ethnicity With the Risk of Cancer Death Among Persons With Second Primary Cancers (SPCs) **eFigure 4.** Association of Race and Ethnicity With the Risk of Cardiovascular Death Among Persons With Second Primary Cancers (SPCs) **eFigure 5.** Associations of Race and Ethnicity With the Risk of Cancer or Cardiovascular Death by Participants’ Characteristics Among Persons With Second Primary Cancers (SPCs)

Supplement 2.
Data Sharing Statement
