# Supplementary material for: Racial and Ethnic Disparities in Survival Among People With Second Primary Cancer in the US
Source: JAMA Netw Open. 2023 Aug 4;6(8):e2327429. doi: 10.1001/jamanetworkopen.2023.27429 (PMC10403787; doi:10.1001/jamanetworkopen.2023.27429)
Supplement: Supplement 1. — eTable 1. Participant Selection Process SEER Research Plus Data, 18 Registries (Excl AK), Nov 2020 Sub (2000-2018) eTable 2. Definition of Cardiovascular Diseases and International Classification of Diseases (ICD)-10 Codes eTable 3. Classification of Prior Cancer Type eTable 4. Definition of Subtypes for Second Primary Cancers of the Female Breast, Lung and Bronchus, Corpus Uteri, and Non-Hodgkin Lymphoma eTable 5. Distribution of Treatment Receipt for Second Primary Cancer by Race and Ethnicity eTable 6. Associations of Race and Ethnicity With the Risk of Cancer Death Among Persons With Second Primary Cancers (SPCs) (Reference: White) eTable 7. Associations of Race and Ethnicity With the Risk of Cardiovascular Death Among Persons With Second Primary Cancers (SPCs) (Reference: White) eTable 8. Association of Race and Ethnicity With the Risk of Cancer or Cardiovascular Death According to Second Primary Cancer (SPC) Subtype (Reference: White) eTable 9. Association of Race and Ethnicity With the Risk of Cancer or Cardiovascular Death in the Fine-Gray Model Among Persons With Second Primary Cancer (SPCs) eTable 10. Association of Race and Ethnicity With the Risk of Cancer or Cardiovascular Death in Cox Proportional Hazards Models Using Age as a Time-Scale Among Persons With Second Primary Cancers (SPCs) eFigure 1. Distribution of (A) County-Level Household Income, (B) County-Level Urbanicity, (C) Stage at Second Primary Cancer Diagnosis, and (D) Second Primary Cancer Subtype by Race and Ethnicity According to Second Primary Cancer Type eFigure 2. Five-Year, Age-Standardized Relative Survival of Second Primary Cancers by Race and Ethnicity, Overall and by Stage eFigure 3. Association of Race and Ethnicity With the Risk of Cancer Death Among Persons With Second Primary Cancers (SPCs) eFigure 4. Association of Race and Ethnicity With the Risk of Cardiovascular Death Among Persons With Second Primary Cancers (SPCs) eFigure 5. Associations of Race and Ethnicity With the Risk [file jamanetwopen-e2327429-s001.pdf]

Supplemental Online Content

Sung H, Nisotel L, Sedeta E, Islami F, Jemal A. Racial and ethnic disparities in survival among people with second primary cancer in the US. *JAMA Netw Open*. 2023;6(8):e2327429. doi:10.1001/jamanetworkopen.2023.27429

eTable 1. Participant Selection Process SEER Research Plus Data, 18 Registries (Excl AK), Nov 2020 Sub (2000-2018)

eTable 2. Definition of Cardiovascular Diseases and International Classification of Diseases (ICD)-10 Codes

eTable 3. Classification of Prior Cancer Type

eTable 4. Definition of Subtypes for Second Primary Cancers of the Female Breast, Lung and Bronchus, Corpus Uteri, and Non-Hodgkin Lymphoma

eTable 5. Distribution of Treatment Receipt for Second Primary Cancer by Race and Ethnicity

eTable 6. Associations of Race and Ethnicity With the Risk of Cancer Death Among Persons With Second Primary Cancers (SPCs) (Reference: White)

eTable 7. Associations of Race and Ethnicity With the Risk of Cardiovascular Death Among Persons With Second Primary Cancers (SPCs) (Reference: White)

eTable 8. Association of Race and Ethnicity With the Risk of Cancer or Cardiovascular Death According to Second Primary Cancer (SPC) Subtype (Reference: White)

eTable 9. Association of Race and Ethnicity With the Risk of Cancer or Cardiovascular Death in the Fine-Gray Model Among Persons With Second Primary Cancer (SPCs)

eTable 10. Association of Race and Ethnicity With the Risk of Cancer or Cardiovascular Death in Cox Proportional Hazards Models Using Age as a Time-Scale Among Persons With Second Primary Cancers (SPCs)

eFigure 1. Distribution of (A) County-Level Household Income, (B) County-Level Urbanicity, (C) Stage at Second Primary Cancer Diagnosis, and (D) Second Primary Cancer Subtype by Race and Ethnicity According to Second Primary Cancer Type

eFigure 2. Five-Year, Age-Standardized Relative Survival of Second Primary Cancers by Race and Ethnicity, Overall and by Stage

eFigure 3. Association of Race and Ethnicity With the Risk of Cancer Death Among Persons With Second Primary Cancers (SPCs)

© 2023 Sung H et al. *JAMA Network Open*.

eFigure 4. Association of Race and Ethnicity With the Risk of Cardiovascular Death Among Persons With Second Primary Cancers (SPCs)
eFigure 5. Associations of Race and Ethnicity With the Risk of Cancer or Cardiovascular Death by Participants' Characteristics Among Persons With Second Primary Cancers (SPCs)

This supplemental material has been provided by the authors to give readers additional information about their work.

© 2023 Sung H et al. *JAMA Network Open*.

eTable 1. Participant Selection Process SEER Research Plus Data, 18 Registries (Excl AK), Nov 2020 Sub (2000-2018)

Number of total records		8,658,035	
Inclusion criteria	Exclusion criteria	Number of people excluded in each step	Remaining number of survivors after exclusion
People who survived their first primary cancer (sequence number 0 or 1)	Non-index cancers (Cancers that are not first primary (sequence number 0 or 1))	1,828,890	6,829,145
Number of people with malignant cancer	Non-malignant cancers	500,711	6,328,434
People who were not identified with death certificate or autopsy only	Death Certificate or Autopsy Only	82,088	6,246,346
People with known Age Records	Unknown age	537	6,245,809
First primary cancer dx age: 20+; Diagnosis years, 2000-2013;	First primary cancer diagnosis age<21 years; First primary cancer diagnosis years not from 2000-2013; Race and ethnicity unknown records	1,891,328	4,354,481
those with an index record start date after the study cutoff date	First primary cancer diagnosis date after the study cutoff date	49,370	4,305,111
those with a Date of Last Contact on Index Record within Latency exclusion period	Survivors with a date of last contact within latency exclusion period (2 months)	308,509	3,996,602
those with an exit point prior to their entry point	Survivors with an exit point prior to their entry point	62,777	3,933,825
Survivors who developed one of top 10 most common second primary cancers in men or women[a]		3700986	232,839
Survival month unknown		1503	231,336
Prostate cancer survivors who developed second prostate cancer[b]		46	231,290
Survivors of American Indians or Alaskan Natives		983	230,307

[a]Lung and Bronchus, Breast, Colon and Rectum, Prostate, Urinary Bladder, Non-Hodgkin Lymphoma, Melanoma of the skin, Kidney and Renal Pelvis, Oral Cavity and Pharynx, Pancreas, Stomach, Thyroid, Corpus Uteri, not otherwise specified
[b]Considered coding errors

© 2023 Sung H et al. *JAMA Network Open*.

eTable 2. Definition of Cardiovascular Diseases and International Classification of Diseases (ICD)-10 Codes

Non-Neoplasm Causes of Death	ICD-10 (1999+)	Reference
Diseases of Heart	I00-I09, I11, I13, I20-I51	
Hypertension without Heart Disease	I10, I12	SEER Cause of Death Recode 1969+ (03/01/2018) https://seer.cancer.gov/codrecode/1969_d03012018/index.html
Cerebrovascular Diseases	I60-I69	
Atherosclerosis	I70	
Aortic Aneurysm and Dissection	I71	
Other Diseases of Arteries, Arterioles, Capillaries	I72-I78	

© 2023 Sung H et al. *JAMA Network Open*.

eTable 3. Classification of Prior Cancer Type

Group	Prior cancer type (tumor sequence defined as "1", i.e., First primary cancer among those with multiple primary cancer)
Bone/Soft Tissue	Bones and Joints, Soft Tissue including Heart
Urinary	Urinary Bladder, Kidney and Renal Pelvis, Ureter, Other Urinary Organs
Skin/Eye	Melanoma of the Skin, Other Non-Epithelial Skin, Eye and Orbit
Respiratory	Lung and Bronchus, Larynx, Nose, Nasal Cavity and Middle Ear, Trachea, Mediastinum and Other Respiratory Organs, Pleura
Oral cavity and pharynx	Oral cavity and pharynx
Other	Miscellaneous, Mesothelioma, Kaposi Sarcoma
Genital	Prostate; Corpus Uteri, Not Otherwise Specified; Ovary; Cervix Uteri; Vagina and Vulva; Testis; Penis
Endocrine	Thyroid, Other Endocrine including Thymus
Digestive	Colon and Rectum, Stomach, Esophagus, Liver and Intrahepatic Bile Duct, Small Intestine, Pancreas, Anus, Anal Canal and Anorectum, Gallbladder and Other Biliary, Retroperitoneum, Peritoneum, Omentum and Mesentery, Other Digestive Organs
Female breast	Female breast
Brain and Other Nervous System	Brain and Other Nervous System
Blood	Non-Hodgkin Lymphoma, Myeloma, Leukemia, Hodgkin Lymphoma

© 2023 Sung H et al. *JAMA Network Open*.

eTable 4. Definition of Subtypes for Second Primary Cancers of the Female Breast, Lung and Bronchus, Corpus Uteri, NOS, and Non-Hodgkin Lymphoma

Site group	Subtype	SEER variables	Definition
Female breast	Hormone receptor-positive	ER Status Recode Female breast Cancer (1990+); PR Status Recode Female breast Cancer (1990+)	(estrogen receptor positive or borderline) OR (progesterone receptor positive or borderline)
	Hormone receptor-negative	ER Status Recode Female breast Cancer (1990+); PR Status Recode Female breast Cancer (1990+)	estrogen receptor negative AND progesterone receptor positive
	Hormone receptor-unknown	ER Status Recode Female breast Cancer (1990+); PR Status Recode Female breast Cancer (1990+)	
Non-Hodgkin Lymphoma	Chronic lymphocytic leukemia/small lymphocytic lymphoma	ICD-O-3 Histology (Type)	9823/3, 9670/3
	Marginal zone lymphoma	ICD-O-3 Histology (Type)	9699/3, 9689/3
	Follicular lymphoma	ICD-O-3 Histology (Type)	9698/3, 9695/3, 9691/3, 9690/3, 9597/3
	Diffuse large B-cell lymphoma	ICD-O-3 Histology (Type)	9738/3, 9737/3, 9735/3, 9712/3, 9688/3, 9684/3, 9680/3, 9679/3, 9678/3
Lung and Bronchus	Non-small cell squamous cell carcinoma	ICD-O-3 Histology (Type)	8051-8052, 8070-8076, 8078, 8083-8084, 8090, 8094, 8120, 8123
	Non-small cell adenocarcinoma	ICD-O-3 Histology (Type)	8015, 8050, 8140-8141, 8143-8145, 8147, 8190, 8201, 8211, 8250-8255, 8260, 8290, 8310, 8320, 8323, 8333, 8401,8440, 8470-8471, 8480-8481, 8490, 8503, 8507, 8550, 8570-8572, 8574, 8576
	Large cell carcinoma	ICD-O-3 Histology (Type)	8012-8014, 8021, 8034, 8082
	Non-small cell cancer, not otherwise specified	ICD-O-3 Histology (Type)	8003-8004, 8022, 8030-8033, 8035, 8200, 8240-8241, 8243-8246, 8249, 8430, 8525, 8560, 8562, 8575, 8046
	Small cell carcinoma	ICD-O-3 Histology (Type)	8002, 8041-8045
	Other		
Corpus uteri, Not Otherwise Specified	Nonendometrioid	ICD-O-3 Histology (Type)	8255 , 8310 , 8323 , 8441 , 8460 , 8461 , 8950 , 8951 , 8980 , 8981

© 2023 Sung H et al. *JAMA Network Open*.

	Endometrioid	ICD-O-3 Histology (Type)	8050 , 8141, 8210 , 8211 , 8260 , 8261 , 8262 , 8263 , 8380 , 8381 , 8382 , 8383 , 8440 , 8470 , 8471 , 8480 , 8481 , 8490 , 8560 , 8570 , 8571 , 8140
	Sarcomas	ICD-O-3 Histology (Type)	8800 , 8801 , 8802 , 8803 , 8804 , 8805 , 8810 , 8811 , 8814 , 8840 , 8850 , 8853 , 8855 , 8858 , 8860 , 8890 , 8891 , 8895 , 8896 , 8900 , 8901 , 8902 , 8910 , 8912 , 8920 , 8930 , 8931 , 8933 , 8935 , 8936 , 9120 , 9180 , 9220 , 9240 , 9260
	Other	ICD-O-3 Histology (Type)	All other codes
Colorectum	Colon excluding Rectum	ICD-O-3 Site	C180, C181, C182, C183, C184, C185, C186, C187, C188-C189, C260
	Rectum and Rectosigmoid Junction	ICD-O-3 Site	C199, C209

© 2023 Sung H et al. *JAMA Network Open*.

eTable 5. Distribution of Treatment Receipt for Second Primary Cancer by Race and Ethnicity

Second primary cancer type	Race and ethnicity[a]	Total, n	Surgery, row %			Radiotherapy, row %		Chemotherapy, row %	
			Performed	Not performed	Unknown	Yes	None/ Unknown	No/Unknown	Yes
Female breast	Asian or Pacific Islander	1786	89.7	9.2	1.1	32.5	67.5	66.9	33.2
	Black	3300	83.3	15.4	1.3	28.7	71.3	61.9	38.1
	Hispanic	2496	85.9	13.0	1.0	29.6	70.4	64.7	35.3
	White	22253	91.0	8.4	0.6	35.9	64.1	73.9	26.1
Colon and rectum	Asian or Pacific Islander	1560	81.3	17.7	1.0	9.0	91.0	74.8	25.2
	Black	3453	78.6	20.6	0.8	7.3	92.7	73.3	26.7
	Hispanic	2135	80.1	19.1	0.8	10.2	89.8	72.0	28.0
	White	21275	83.4	15.8	0.9	8.9	91.1	74.5	25.5
Corpus uteri, Not Otherwise Specified	Asian or Pacific Islander	369	92.4	7.1	0.5	18.7	81.3	75.3	24.7
	Black	488	81.8	17.4	0.8	26.4	73.6	74.4	25.6
	Hispanic	427	86.9	12.7	0.5	22.7	77.3	79.6	20.4
	White	4104	89.1	10.2	0.7	23.8	76.2	83.1	16.9
Kidney and renal pelvis	Asian or Pacific Islander	443	79.9	18.7	1.4	2.9	97.1	95.3	4.7
	Black	1609	79.6	19.3	1.1	1.9	98.1	96.6	3.4
	Hispanic	1091	82.4	17.0	0.6	2.2	97.8	94.6	5.4
	White	9169	81.0	18.1	0.9	3.0	97.0	94.1	5.9
Lung and bronchus	Asian or Pacific Islander	1958	33.2	65.2	1.6	28.3	71.7	65.5	34.5
	Black	5196	23.8	75.4	0.8	35.7	64.3	62.2	37.8
	Hispanic	2242	28.0	71.1	0.9	28.6	71.5	67.2	32.8
	White	38670	30.9	68.1	1.0	32.5	67.5	65.1	34.9
Melanoma of the skin	Asian or Pacific Islander	92	87.0	13.0	0.0	2.2	97.8	96.7	3.3
	Black	104	92.3	7.7	0.0	1.0	99.0	98.1	1.9
	Hispanic	334	87.7	11.7	0.6	2.7	97.3	97.3	2.7
	White	15901	92.5	7.0	0.5	2.5	97.6	98.1	1.9
Non-Hodgkin lymphoma	Asian or Pacific Islander	679	24.5	73.8	1.8	15.2	84.8	48.3	51.7
	Black	1098	20.8	78.4	0.8	9.1	90.9	59.7	40.4
	Hispanic	1218	23.4	76.2	0.4	10.0	90.0	51.7	48.3
	White	13353	22.6	76.7	0.7	10.9	89.1	57.0	43.1
Oral cavity and pharynx	Asian or Pacific Islander	399	60.9	37.6	1.5	44.6	55.4	69.7	30.3
	Black	752	45.5	53.3	1.2	52.0	48.0	63.6	36.4

© 2023 Sung H et al. *JAMA Network Open*.

	Hispanic	461	58.6	40.1	1.3	40.1	59.9	73.5	26.5
	White	6611	61.1	37.9	1.0	45.6	54.4	71.5	28.5
Pancreas	Asian or Pacific Islander	392	20.2	78.3	1.5	13.8	86.2	61.0	39.0
	Black	911	14.1	84.9	1.1	14.6	85.4	60.7	39.3
	Hispanic	537	18.3	80.8	0.9	10.2	89.8	62.6	37.4
	White	5864	18.9	79.8	1.3	14.6	85.4	58.4	41.6
Prostate	Asian or Pacific Islander	823	34.8	62.6	2.7	30.6	69.4	99.4	0.6
	Black	2702	29.1	69.7	1.3	34.5	65.5	99.3	0.7
	Hispanic	1530	39.8	59.0	1.2	27.5	72.6	99.3	0.7
	White	20059	41.8	56.6	1.6	29.0	71.0	99.2	0.8
Stomach	Asian or Pacific Islander	566	55.3	42.4	2.3	14.5	85.5	72.3	27.7
	Black	771	45.7	53.6	0.8	13.4	86.6	69.4	30.6
	Hispanic	581	42.5	56.5	1.0	12.2	87.8	67.6	32.4
	White	3227	43.7	54.4	1.9	21.1	78.9	65.4	34.6
Thyroid	Asian or Pacific Islander	511	89.8	9.6	0.6	44.6	55.4	97.7	2.4
	Black	379	89.5	9.8	0.8	36.2	63.9	98.4	1.6
	Hispanic	645	90.9	8.4	0.8	44.7	55.4	98.9	1.1
	White	4400	92.1	7.2	0.7	40.1	59.9	98.6	1.5
Urinary bladder	Asian or Pacific Islander	716	90.2	8.5	1.3	4.6	95.4	84.9	15.1
	Black	1314	88.0	11.9	0.2	6.9	93.2	83.9	16.1
	Hispanic	1143	88.2	10.9	1.0	4.1	95.9	84.1	15.9
	White	18273	90.6	8.9	0.4	4.4	95.6	83.2	16.8

[a]All race categories are exclusive of Hispanic ethnicity.

© 2023 Sung H et al. *JAMA Network Open*.

eTable 6. Associations of Race and Ethnicity[a] With the Risk of Cancer Death Among Persons With Second Primary Cancers (SPCs) (Reference: White)

Race and ethnicity	Second primary cancer	Variables adjusted in the model[b]			Proportional reduction in the hazard ratio from Model 1 to Model 3[c]
		Model1<br>Age at diagnosis, year of diagnosis, sex, prior cancer type, prior cancer stage<br>Hazard ratio (95% CI)	Model2<br>Model 1+county-level median household income, urbanicity<br>Hazard ratio (95% CI)	Model3<br>Model 2+SPC stage, treatment for SPC (surgery, radiotherapy, chemotherapy), SPC subtype<br>Hazard ratio (95% CI)	
Asian or Pacific Islander	All second primary cancers	0.93 (0.9 to 0.96)	0.98 (0.95 to 1.01)	0.9 (0.86 to 0.93)	c
Asian or Pacific Islander	Female breast	0.91 (0.83 to 1)	0.95 (0.86 to 1.05)	0.87 (0.79 to 0.96)	c
Asian or Pacific Islander	Colon and Rectum	0.91 (0.84 to 0.98)	0.93 (0.86 to 1.01)	0.91 (0.84 to 0.99)	c
Asian or Pacific Islander	Corpus Uteri, NOS	1.18 (0.98 to 1.42)	1.2 (0.99 to 1.45)	1.23 (1 to 1.52)	-31.6
Asian or Pacific Islander	Kidney and Renal Pelvis	1.01 (0.86 to 1.18)	1.09 (0.93 to 1.28)	0.97 (0.82 to 1.14)	c
Asian or Pacific Islander	Lung and Bronchus	0.87 (0.83 to 0.92)	0.92 (0.88 to 0.98)	0.84 (0.8 to 0.89)	c
Asian or Pacific Islander	Melanoma of the Skin	1.93 (1.42 to 2.62)	2.04 (1.5 to 2.77)	1.75 (1.28 to 2.38)	19.8
Asian or Pacific Islander	Non-Hodgkin Lymphoma	1.13 (1.01 to 1.27)	1.18 (1.06 to 1.33)	1.13 (1 to 1.27)	5.2
Asian or Pacific Islander	Oral cavity and pharynx	0.92 (0.79 to 1.07)	0.96 (0.82 to 1.11)	0.95 (0.81 to 1.1)	c
Asian or Pacific Islander	Pancreas	0.92 (0.82 to 1.03)	0.95 (0.85 to 1.06)	0.93 (0.83 to 1.05)	c
Asian or Pacific Islander	Prostate	0.97 (0.86 to 1.1)	1 (0.88 to 1.13)	1.01 (0.89 to 1.15)	c
Asian or Pacific Islander	Stomach	0.86 (0.77 to 0.96)	0.89 (0.79 to 0.99)	1 (0.89 to 1.12)	c
Asian or Pacific Islander	Thyroid	1.26 (1.04 to 1.54)	1.3 (1.07 to 1.59)	1.18 (0.96 to 1.46)	30.3
Asian or Pacific Islander	Urinary Bladder	0.85 (0.76 to 0.96)	0.89 (0.79 to 1)	0.89 (0.79 to 1.01)	c
Black	All second primary cancers	1.21 (1.18 to 1.23)	1.19 (1.17 to 1.21)	1.08 (1.05 to 1.11)	61.2
Black	Female breast	1.51 (1.42 to 1.6)	1.47 (1.38 to 1.56)	1.19 (1.12 to 1.27)	61.9
Black	Colon and Rectum	1.24 (1.18 to 1.31)	1.24 (1.18 to 1.3)	1.16 (1.1 to 1.22)	36.2
Black	Corpus Uteri, NOS	1.87 (1.63 to 2.15)	1.84 (1.6 to 2.13)	1.49 (1.27 to 1.76)	43.4
Black	Kidney and Renal Pelvis	0.99 (0.9 to 1.09)	0.98 (0.89 to 1.08)	0.96 (0.87 to 1.06)	c
Black	Lung and Bronchus	1.09 (1.06 to 1.13)	1.08 (1.05 to 1.12)	0.98 (0.94 to 1.01)	c
Black	Melanoma of the Skin	1.58 (1.17 to 2.12)	1.52 (1.13 to 2.05)	1.23 (0.91 to 1.66)	60.5
Black	Non-Hodgkin Lymphoma	1.28 (1.17 to 1.4)	1.24 (1.13 to 1.36)	1.23 (1.12 to 1.35)	18.5
Black	Oral cavity and pharynx	1.46 (1.32 to 1.61)	1.44 (1.31 to 1.59)	1.29 (1.16 to 1.43)	37.2
Black	Pancreas	1.15 (1.07 to 1.24)	1.14 (1.05 to 1.23)	1.04 (0.96 to 1.12)	76.5
Black	Prostate	1.17 (1.09 to 1.26)	1.16 (1.08 to 1.25)	1.13 (1.05 to 1.22)	25.3
Black	Stomach	1.04 (0.95 to 1.15)	1.02 (0.93 to 1.13)	1.08 (0.98 to 1.2)	-81.8
Black	Thyroid	1.2 (0.95 to 1.5)	1.19 (0.94 to 1.5)	1.07 (0.84 to 1.37)	64.6

© 2023 Sung H et al. *JAMA Network Open*.

Black	Urinary Bladder	1.38 (1.27 to 1.5)	1.36 (1.25 to 1.47)	1.24 (1.14 to 1.35)	36.1
Hispanic	All second primary cancers	1.1 (1.07 to 1.13)	1.11 (1.09 to 1.14)	1.04 (1 to 1.07)	63.4
Hispanic	Female breast	1.27 (1.18 to 1.37)	1.27 (1.18 to 1.37)	1.09 (1.01 to 1.18)	65.7
Hispanic	Colon and Rectum	1.09 (1.03 to 1.17)	1.11 (1.04 to 1.18)	1.11 (1.04 to 1.18)	-11.7
Hispanic	Corpus Uteri, NOS	1.34 (1.14 to 1.59)	1.34 (1.14 to 1.59)	1.14 (0.94 to 1.38)	60.2
Hispanic	Kidney and Renal Pelvis	0.98 (0.88 to 1.1)	1.01 (0.91 to 1.13)	0.94 (0.84 to 1.05)	[c]
Hispanic	Lung and Bronchus	1.02 (0.97 to 1.07)	1.04 (0.99 to 1.09)	0.95 (0.9 to 1)	[c]
Hispanic	Melanoma of the Skin	1.46 (1.21 to 1.76)	1.48 (1.23 to 1.79)	1.39 (1.14 to 1.69)	15.6
Hispanic	Non-Hodgkin Lymphoma	1.27 (1.16 to 1.38)	1.26 (1.16 to 1.38)	1.19 (1.09 to 1.31)	27.2
Hispanic	Oral cavity and pharynx	1.22 (1.08 to 1.39)	1.24 (1.09 to 1.41)	1.19 (1.04 to 1.36)	14.5
Hispanic	Pancreas	1.04 (0.95 to 1.15)	1.05 (0.95 to 1.15)	1 (0.91 to 1.1)	[c]
Hispanic	Prostate	0.98 (0.89 to 1.08)	0.98 (0.89 to 1.08)	0.95 (0.86 to 1.05)	[c]
Hispanic	Stomach	1.04 (0.93 to 1.16)	1.03 (0.92 to 1.15)	1.01 (0.9 to 1.13)	69.4
Hispanic	Thyroid	1.2 (0.99 to 1.45)	1.22 (1.01 to 1.48)	1.13 (0.93 to 1.38)	34.0
Hispanic	Urinary Bladder	1.12 (1.02 to 1.22)	1.13 (1.03 to 1.24)	1.09 (0.99 to 1.19)	23.5

[a]All race categories are exclusive of Hispanic ethnicity.

[b]Covariates sequentially adjusted in Cox proportional hazards models include: sex (male, female), prior cancer type/system (eTable2 for details), prior cancer stage (localized, regional, distant, unknown/unstaged/blank) and year (2000-2004, 2005-2009, 2010-2013) and age (5-year interval) at second primary cancer diagnosis, county-level household income (<$35,000-$59,000, $60,000-$74,999, $75,000+, unknown), county-level urbanity (large metropolitan, small metropolitan, nonmetropolitan, unknown), SPC type (only for all SPCs combined), SPC stage, SPC subtype (eTable3 for details), first course of SPC treatment including receipt of surgery, receipt of radiotherapy, and receipt of chemotherapy for SPC.

[c]Calculated using the formular only when both HR_{Model1} and HR_{Model3} were more than 1: $[HR_{Model1} – HR_{Model3}] \div [HR_{Model1}] \times 100$

© 2023 Sung H et al. *JAMA Network Open*.

eTable 7. Associations of Race and Ethnicity[a] With the Risk of Cardiovascular Death Among Persons With Second Primary Cancers (SPCs) (Reference: White)

Race and ethnicity	Second primary cancer	Variables adjusted in the model[b]			Proportional reduction in the hazard ratio from Model 1 to Model 3[c]
		Model1<br>Age at diagnosis, year of diagnosis, sex, prior cancer type, prior cancer stage<br>Hazard ratio (95% CI)	Model2<br>Model 1+county-level median household income, urbanicity<br>Hazard ratio (95% CI)	Model3<br>Model 2+SPC stage, treatment for SPC (surgery, radiotherapy, chemotherapy), SPC subtype<br>Hazard ratio (95% CI)	
Asian or Pacific Islander	All second primary cancers	0.75 (0.69 to 0.81)	0.78 (0.72 to 0.85)	0.78 (0.69 to 0.87)	c
Asian or Pacific Islander	Female breast	0.74 (0.58 to 0.94)	0.78 (0.61 to 0.99)	0.73 (0.57 to 0.94)	c
Asian or Pacific Islander	Colon and Rectum	0.73 (0.61 to 0.88)	0.76 (0.63 to 0.91)	0.73 (0.6 to 0.88)	c
Asian or Pacific Islander	Corpus Uteri, NOS	0.83 (0.47 to 1.47)	0.89 (0.5 to 1.59)	0.88 (0.48 to 1.61)	c
Asian or Pacific Islander	Kidney and Renal Pelvis	0.79 (0.54 to 1.13)	0.82 (0.57 to 1.19)	0.81 (0.56 to 1.17)	c
Asian or Pacific Islander	Lung and Bronchus	0.83 (0.68 to 1.02)	0.88 (0.72 to 1.08)	0.86 (0.69 to 1.06)	c
Asian or Pacific Islander	Melanoma of the Skin	0.86 (0.41 to 1.81)	0.9 (0.43 to 1.9)	0.9 (0.43 to 1.91)	c
Asian or Pacific Islander	Non-Hodgkin Lymphoma	0.73 (0.52 to 1.02)	0.75 (0.54 to 1.05)	0.84 (0.59 to 1.19)	c
Asian or Pacific Islander	Oral cavity and pharynx	0.99 (0.66 to 1.49)	1.05 (0.7 to 1.59)	1.04 (0.69 to 1.59)	c
Asian or Pacific Islander	Pancreas	1.23 (0.67 to 2.25)	1.27 (0.69 to 2.34)	1.29 (0.69 to 2.4)	-28.2
Asian or Pacific Islander	Prostate	0.63 (0.49 to 0.82)	0.66 (0.51 to 0.86)	0.65 (0.5 to 0.85)	c
Asian or Pacific Islander	Stomach	0.75 (0.52 to 1.08)	0.81 (0.56 to 1.17)	0.9 (0.62 to 1.32)	c
Asian or Pacific Islander	Thyroid	0.31 (0.14 to 0.71)	0.32 (0.14 to 0.73)	0.3 (0.13 to 0.69)	c
Asian or Pacific Islander	Urinary Bladder	0.74 (0.59 to 0.92)	0.76 (0.61 to 0.96)	0.75 (0.59 to 0.94)	c
Black	All second primary cancers	1.41 (1.34 to 1.49)	1.39 (1.32 to 1.46)	1.3 (1.21 to 1.4)	26.7
Black	Female breast	1.56 (1.35 to 1.79)	1.54 (1.34 to 1.78)	1.47 (1.26 to 1.7)	16.5
Black	Colon and Rectum	1.26 (1.12 to 1.42)	1.26 (1.12 to 1.43)	1.23 (1.09 to 1.39)	13.4
Black	Corpus Uteri, NOS	1.43 (0.96 to 2.13)	1.36 (0.91 to 2.04)	1.35 (0.86 to 2.11)	19.4
Black	Kidney and Renal Pelvis	1.63 (1.38 to 1.93)	1.59 (1.34 to 1.89)	1.51 (1.27 to 1.79)	19.4
Black	Lung and Bronchus	1.3 (1.14 to 1.47)	1.28 (1.12 to 1.45)	1.19 (1.05 to 1.36)	34.5
Black	Melanoma of the Skin	1.17 (0.58 to 2.37)	1.14 (0.56 to 2.32)	1.14 (0.56 to 2.31)	18.9
Black	Non-Hodgkin Lymphoma	1.49 (1.21 to 1.85)	1.44 (1.16 to 1.78)	1.39 (1.11 to 1.74)	20.7
Black	Oral cavity and pharynx	1.5 (1.12 to 2.01)	1.5 (1.12 to 2.01)	1.53 (1.13 to 2.06)	-5.2
Black	Pancreas	1.8 (1.17 to 2.75)	1.79 (1.16 to 2.76)	1.64 (1.06 to 2.55)	19.2

© 2023 Sung H et al. *JAMA Network Open*.

Black	Prostate	1.24 (1.08 to 1.42)	1.22 (1.06 to 1.4)	1.2 (1.04 to 1.37)	17.4
Black	Stomach	1.45 (1.08 to 1.95)	1.33 (0.98 to 1.8)	1.47 (1.08 to 2)	-3.8
Black	Thyroid	1.7 (1.12 to 2.57)	1.61 (1.06 to 2.46)	1.57 (1.01 to 2.43)	18.2
Black	Urinary Bladder	1.48 (1.27 to 1.73)	1.47 (1.26 to 1.73)	1.47 (1.26 to 1.72)	2.1
Hispanic	All second primary cancers	0.9 (0.84 to 0.96)	0.9 (0.84 to 0.97)	0.89 (0.8 to 0.98)	[c]
Hispanic	Female breast	0.87 (0.71 to 1.08)	0.88 (0.71 to 1.08)	0.85 (0.69 to 1.06)	[c]
Hispanic	Colon and Rectum	0.84 (0.72 to 1)	0.85 (0.72 to 1.01)	0.84 (0.71 to 1)	[c]
Hispanic	Corpus Uteri, NOS	0.86 (0.49 to 1.53)	0.85 (0.48 to 1.5)	0.81 (0.44 to 1.51)	[c]
Hispanic	Kidney and Renal Pelvis	0.82 (0.63 to 1.07)	0.82 (0.63 to 1.07)	0.82 (0.63 to 1.07)	[c]
Hispanic	Lung and Bronchus	0.91 (0.74 to 1.11)	0.93 (0.76 to 1.13)	0.84 (0.68 to 1.03)	[c]
Hispanic	Melanoma of the Skin	1.08 (0.72 to 1.61)	1.06 (0.71 to 1.59)	1 (0.67 to 1.5)	[c]
Hispanic	Non-Hodgkin Lymphoma	1.16 (0.93 to 1.45)	1.15 (0.92 to 1.44)	1.15 (0.91 to 1.45)	6.3
Hispanic	Oral cavity and pharynx	0.92 (0.61 to 1.38)	0.91 (0.6 to 1.38)	0.91 (0.6 to 1.4)	[c]
Hispanic	Pancreas	1.06 (0.58 to 1.94)	1.08 (0.59 to 1.99)	1.09 (0.59 to 2.01)	-45.9
Hispanic	Prostate	0.91 (0.76 to 1.09)	0.92 (0.77 to 1.1)	0.91 (0.76 to 1.08)	[c]
Hispanic	Stomach	0.67 (0.43 to 1.03)	0.64 (0.42 to 0.99)	0.7 (0.45 to 1.09)	[c]
Hispanic	Thyroid	0.73 (0.43 to 1.24)	0.7 (0.41 to 1.2)	0.69 (0.4 to 1.19)	[c]
Hispanic	Urinary Bladder	0.92 (0.76 to 1.1)	0.92 (0.77 to 1.11)	0.93 (0.77 to 1.12)	[c]

[a]All race categories are exclusive of Hispanic ethnicity.

[b]Covariates sequentially adjusted in Cox proportional hazards models include: sex (male, female), prior cancer type/system (eTable2 for details), prior cancer stage (localized, regional, distant, unknown/unstaged/blank) and year (2000-2004, 2005-2009, 2010-2013) and age (5-year interval) at second primary cancer diagnosis, county-level household income (<$35,000-$59,000, $60,000-$74,999, $75,000+, unknown), county-level urbanity (large metropolitan, small metropolitan, nonmetropolitan, unknown), SPC type (only for all SPCs combined), SPC stage, SPC subtype (eTable3 for details), first course of SPC treatment including receipt of surgery, receipt of radiotherapy, and receipt of chemotherapy for SPC.

[c]Calculated using the formular only when both HR_{Model1} and HR_{Model3} were more than 1: $[HR_{Model1} – HR_{Model3}] ÷ [HR_{Model1}] × 100$

© 2023 Sung H et al. *JAMA Network Open*.

eTable 8. Association of Race and Ethnicity With the Risk of Cancer or Cardiovascular Death According to Second Primary Cancer (SPC) Subtype (Reference: White)

Second primary cancer	Second primary cancer subtype	Race and Ethnicity[a]	HR (95% CI) for cancer death[b]	HR (95% CI) for cardiovascular death[b]
Female breast	Hormone receptor-negative	Asian or Pacific Islander	0.77 (0.63 to 0.95)	0.72 (0.4 to 1.3)
Female breast	Hormone receptor-positive	Asian or Pacific Islander	0.94 (0.83 to 1.06)	0.78 (0.59 to 1.03)
Female breast	Hormone receptor-negative	Black	1.31 (1.17 to 1.46)	2.08 (1.53 to 2.83)
Female breast	Hormone receptor-positive	Black	1.41 (1.29 to 1.53)	1.39 (1.16 to 1.66)
Female breast	Hormone receptor-negative	Hispanics	1.16 (1.01 to 1.34)	1.1 (0.67 to 1.81)
Female breast	Hormone receptor-positive	Hispanics	1.26 (1.14 to 1.39)	0.8 (0.61 to 1.03)
Colon and Rectum	Colon	Asian or Pacific Islander	0.85 (0.77 to 0.93)	0.66 (0.53 to 0.83)
Colon and Rectum	Rectum	Asian or Pacific Islander	1 (0.88 to 1.13)	0.95 (0.69 to 1.3)
Colon and Rectum	Colon	Black	1.28 (1.2 to 1.35)	1.29 (1.13 to 1.47)
Colon and Rectum	Rectum	Black	1.18 (1.07 to 1.3)	1.15 (0.88 to 1.51)
Colon and Rectum	Colon	Hispanics	1.09 (1.01 to 1.18)	0.85 (0.7 to 1.04)
Colon and Rectum	Rectum	Hispanics	1.09 (0.98 to 1.21)	0.83 (0.6 to 1.15)
Corpus Uteri, NOS	Endometrioid	Asian or Pacific Islander	1 (0.75 to 1.31)	0.74 (0.36 to 1.53)
Corpus Uteri, NOS	Nonendometrioid	Asian or Pacific Islander	1.33 (0.99 to 1.78)	1.1 (0.4 to 3.06)
Corpus Uteri, NOS	Other	Asian or Pacific Islander	0.49 (0.23 to 1.05)	0.55 (0.06 to 5.33)
Corpus Uteri, NOS	Sarcomas	Asian or Pacific Islander	1.81 (0.85 to 3.87)	[c]
Corpus Uteri, NOS	Endometrioid	Black	1.9 (1.53 to 2.35)	1.45 (0.85 to 2.47)
Corpus Uteri, NOS	Nonendometrioid	Black	1.37 (1.11 to 1.7)	1.43 (0.72 to 2.85)
Corpus Uteri, NOS	Other	Black	0.6 (0.34 to 1.05)	1.12 (0.27 to 4.63)
Corpus Uteri, NOS	Sarcomas	Black	1.8 (1.09 to 2.96)	[c]
Corpus Uteri, NOS	Endometrioid	Hispanics	1.39 (1.1 to 1.75)	1.07 (0.57 to 1.99)
Corpus Uteri, NOS	Nonendometrioid	Hispanics	0.98 (0.74 to 1.3)	0.5 (0.12 to 2.11)
Corpus Uteri, NOS	Other	Hispanics	1.2 (0.64 to 2.24)	[c]
Corpus Uteri, NOS	Sarcomas	Hispanics	1.26 (0.66 to 2.42)	[c]
Lung and Bronchus	Non-small cell lung cancer	Asian or Pacific Islander	0.89 (0.84 to 0.95)	0.81 (0.64 to 1.01)
Lung and Bronchus	Other	Asian or Pacific Islander	0.93 (0.78 to 1.1)	1.06 (0.61 to 1.86)
Lung and Bronchus	Small cell lung cancer	Asian or Pacific Islander	0.85 (0.71 to 1.03)	0.9 (0.37 to 2.21)
Lung and Bronchus	Non-small cell lung cancer	Black	1.14 (1.1 to 1.19)	1.25 (1.09 to 1.44)
Lung and Bronchus	Other	Black	0.98 (0.88 to 1.1)	1.76 (1.29 to 2.42)
Lung and Bronchus	Small cell lung cancer	Black	0.97 (0.87 to 1.08)	0.93 (0.51 to 1.68)
Lung and Bronchus	Non-small cell lung cancer	Hispanics	1.02 (0.97 to 1.08)	0.89 (0.71 to 1.11)
Lung and Bronchus	Other	Hispanics	1.16 (1 to 1.34)	0.68 (0.34 to 1.38)

© 2023 Sung H et al. *JAMA Network Open*.

Lung and Bronchus	Small cell lung cancer	Hispanics	0.97 (0.83 to 1.14)	1.77 (0.93 to 3.38)
Non-Hodgkin Lymphoma	Chronic lymphocytic leukemia/small lymphocytic lymphoma	Asian or Pacific Islander	0.5 (0.29 to 0.86)	1.01 (0.47 to 2.13)
Non-Hodgkin Lymphoma	Diffuse large B-cell lymphoma	Asian or Pacific Islander	1.13 (0.95 to 1.33)	0.58 (0.31 to 1.1)
Non-Hodgkin Lymphoma	Follicular lymphoma	Asian or Pacific Islander	0.73 (0.47 to 1.15)	0.27 (0.07 to 1.08)
Non-Hodgkin Lymphoma	Marginal Zone Lymphoma	Asian or Pacific Islander	0.86 (0.54 to 1.39)	0.74 (0.27 to 2)
Non-Hodgkin Lymphoma	Other	Asian or Pacific Islander	1.29 (1.07 to 1.56)	1.03 (0.58 to 1.81)
Non-Hodgkin Lymphoma	Chronic lymphocytic leukemia/small lymphocytic lymphoma	Black	1.21 (1.01 to 1.45)	1.38 (0.97 to 1.97)
Non-Hodgkin Lymphoma	Diffuse large B-cell lymphoma	Black	1.41 (1.2 to 1.65)	1.72 (1.08 to 2.75)
Non-Hodgkin Lymphoma	Follicular lymphoma	Black	1.43 (1.02 to 2)	1.12 (0.49 to 2.55)
Non-Hodgkin Lymphoma	Marginal Zone Lymphoma	Black	1.34 (0.95 to 1.88)	0.85 (0.35 to 2.08)
Non-Hodgkin Lymphoma	Other	Black	1.2 (1.02 to 1.41)	1.81 (1.24 to 2.63)
Non-Hodgkin Lymphoma	Chronic lymphocytic leukemia/small lymphocytic lymphoma	Hispanics	1.24 (0.98 to 1.55)	1.08 (0.64 to 1.82)
Non-Hodgkin Lymphoma	Diffuse large B-cell lymphoma	Hispanics	1.18 (1.03 to 1.34)	1.34 (0.92 to 1.94)
Non-Hodgkin Lymphoma	Follicular lymphoma	Hispanics	0.86 (0.64 to 1.16)	1.04 (0.56 to 1.93)
Non-Hodgkin Lymphoma	Marginal Zone Lymphoma	Hispanics	1.31 (0.94 to 1.83)	0.63 (0.26 to 1.54)
Non-Hodgkin Lymphoma	Other	Hispanics	1.44 (1.23 to 1.69)	1.3 (0.83 to 2.03)

[a]All race categories are exclusive of Hispanic ethnicity.

[b]Adjusted for: sex (male, female), prior cancer type/system (eTable2 for details), prior cancer stage (localized, regional, distant, unknown/unstaged/blank; when appropriate) and year (2000-2004, 2005-2009, 2010-2013) and age (5-year interval) at second primary cancer diagnosis, county-level household income (<$35,000-$59,000, $60,000-$74,999, $75,000+, unknown), county-level urbanity (large metropolitan, small metropolitan, nonmetropolitan, unknown), SPC stage, first course of SPC treatment including receipt of surgery, receipt of radiotherapy, and receipt of chemotherapy for SPC.

[c] Number of SPC cases was too few to be calculated.

© 2023 Sung H et al. *JAMA Network Open*.

eTable 9. Association of Race and Ethnicity[a] With the Risk of Cancer or Cardiovascular Death in the Fine-Gray Model Among Persons With Second Primary Cancers (SPCs)

Cause of death	Variables adjusted in the model[b]		
	Model 1	Model 2	Model 3
Race and ethnicity	Age at diagnosis, year of diagnosis, sex, prior cancer type, prior cancer stage	Model 1+county-level median household income, urbanicity	Model 2+SPC type, SPC stage, treatment for SPC (surgery, radiotherapy, chemotherapy), SPC subtype
	Subdistribution Hazard ratio (95% CI)	Subdistribution Hazard ratio (95% CI)	Subdistribution Hazard ratio (95% CI)
Cancer death			
White	1 (reference)	1 (reference)	1 (reference)
Asian or Pacific Islander	0.95 (0.93 to 0.98)	0.99 (0.96 to 1.02)	0.93 (0.89 to 0.96)
Black	1.16 (1.13 to 1.18)	1.14 (1.12 to 1.17)	1.04 (1.01 to 1.07)
Hispanic	1.10 (1.08 to 1.13)	1.11 (1.09 to 1.14)	1.06 (1.03 to 1.09)
Cardiovascular death			
White			
Asian or Pacific Islander	0.80 (0.73 to 0.86)	0.81 (0.75 to 0.88)	0.83 (0.75 to 0.93)
Black	1.25 (1.19 to 1.32)	1.24 (1.18 to 1.31)	1.24 (1.16 to 1.33)
Hispanic	0.86 (0.80 to 0.92)	0.86 (0.8 to 0.92)	0.86 (0.78 to 0.94)

[a]All race categories are exclusive of Hispanic ethnicity.
[b]Covariates sequentially adjusted in Fine-Gray models include: sex (male, female), prior cancer type/system (eTable2 for details), prior cancer stage (localized, regional, distant, unknown/unstaged/blank) and year (2000-2004, 2005-2009, 2010-2013) and age (5-year interval) at second primary cancer diagnosis, county-level household income (<$35,000-$59,000, $60,000-$74,999, $75,000+, unknown), county-level urbanity (large metropolitan, small metropolitan, nonmetropolitan, unknown), SPC type, SPC stage, SPC subtype (eTable3 for details), first course of SPC treatment including receipt of surgery, receipt of radiotherapy, and receipt of chemotherapy for SPC.

© 2023 Sung H et al. *JAMA Network Open*.

eTable 10. Association of Race and Ethnicity[a] With the Risk of Cancer or Cardiovascular Death in Cox Proportional Hazards Models Using Age as a Time-Scale Among Persons With Second Primary Cancers (SPCs)

Cause of death	Variables adjusted in the model[b]		
	Model 1	Model 2	Model 3
Race and ethnicity	Year of diagnosis, sex, prior cancer type, prior cancer stage	Model 1+county-level median household income, urbanicity	Model 2+SPC type, SPC stage, treatment for SPC (surgery, radiotherapy, chemotherapy), SPC subtype
	Hazard ratio (95% CI)	Hazard ratio (95% CI)	Hazard ratio (95% CI)
Cancer death			
White	1 (reference)	1 (reference)	1 (reference)
Asian or Pacific Islander	0.91 (0.88 to 0.94)	0.96 (0.93 to 0.99)	0.88 (0.84 to 0.92)
Black	1.21 (1.18 to 1.23)	1.19 (1.17 to 1.22)	1.1 (1.07 to 1.13)
Hispanic	1.09 (1.06 to 1.11)	1.1 (1.07 to 1.13)	1.03 (0.99 to 1.07)
Cardiovascular death			
White	1 (reference)	1 (reference)	1 (reference)
Asian or Pacific Islander	0.74 (0.68 to 0.8)	0.77 (0.71 to 0.84)	0.8 (0.71 to 0.9)
Black	1.41 (1.34 to 1.48)	1.39 (1.32 to 1.46)	1.31 (1.22 to 1.41)
Hispanic	0.9 (0.84 to 0.96)	0.9 (0.84 to 0.97)	0.9 (0.81 to 1)

[a]All race categories are exclusive of Hispanic ethnicity.
[b]Covariates sequentially adjusted in Cox proportional hazards models include: sex (male, female), prior cancer type/system (eTable2 for details), prior cancer stage (localized, regional, distant, unknown/unstaged/blank) and year (2000-2004, 2005-2009, 2010-2013) at second primary cancer diagnosis, county-level household income (<$35,000-$59,000, $60,000-$74,999, $75,000+, unknown), county-level urbanity (large metropolitan, small metropolitan, nonmetropolitan, unknown), SPC type, SPC stage, SPC subtype (eTable3 for details), first course of SPC treatment including receipt of surgery, receipt of radiotherapy, and receipt of chemotherapy for SPC.

© 2023 Sung H et al. *JAMA Network Open*.

eFigure 1. Distribution of (A) County-Level Household Income, (B) County-Level Urbanicity, (C) Stage at Second Primary Cancer Diagnosis, and (D) Second Primary Cancer Subtype by Race and Ethnicity According to Second Primary Cancer Type

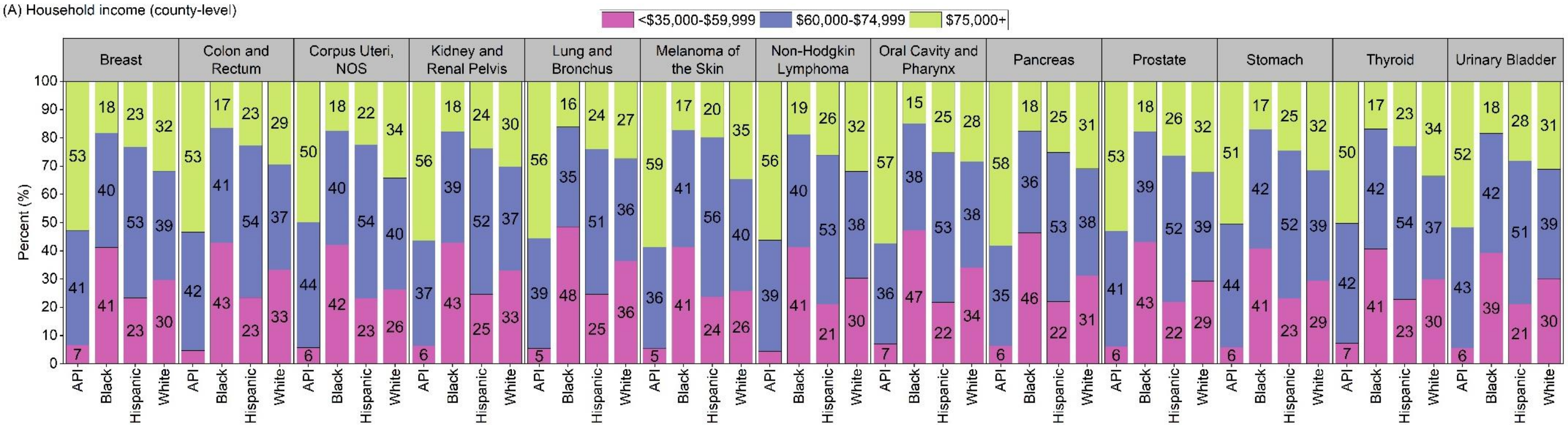

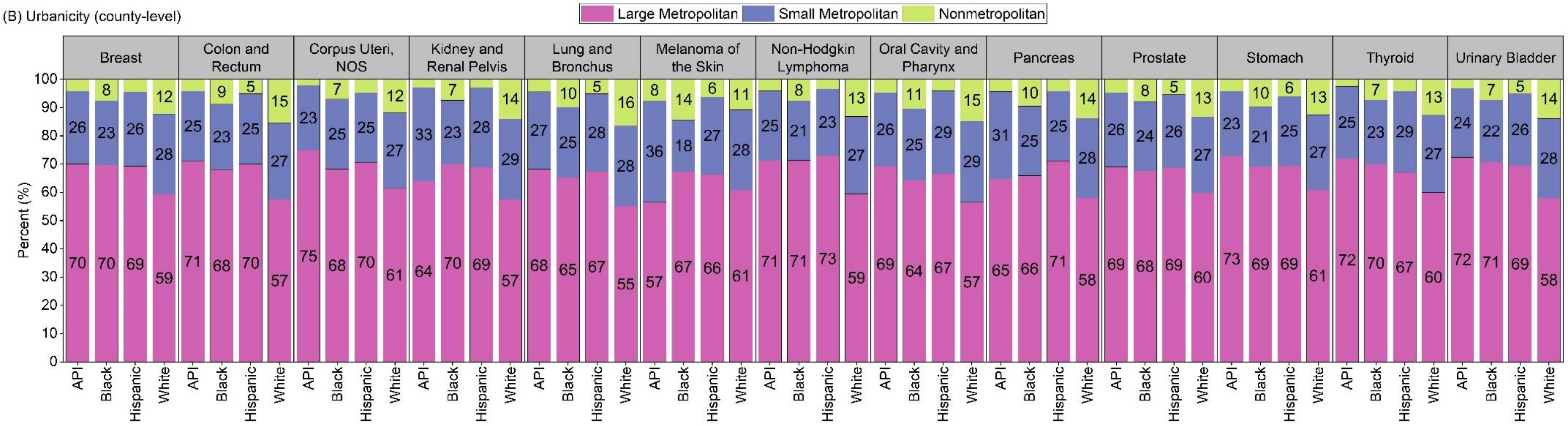

© 2023 Sung H et al. *JAMA Network Open*.

(C) Stage at second primary cancer diagnosis

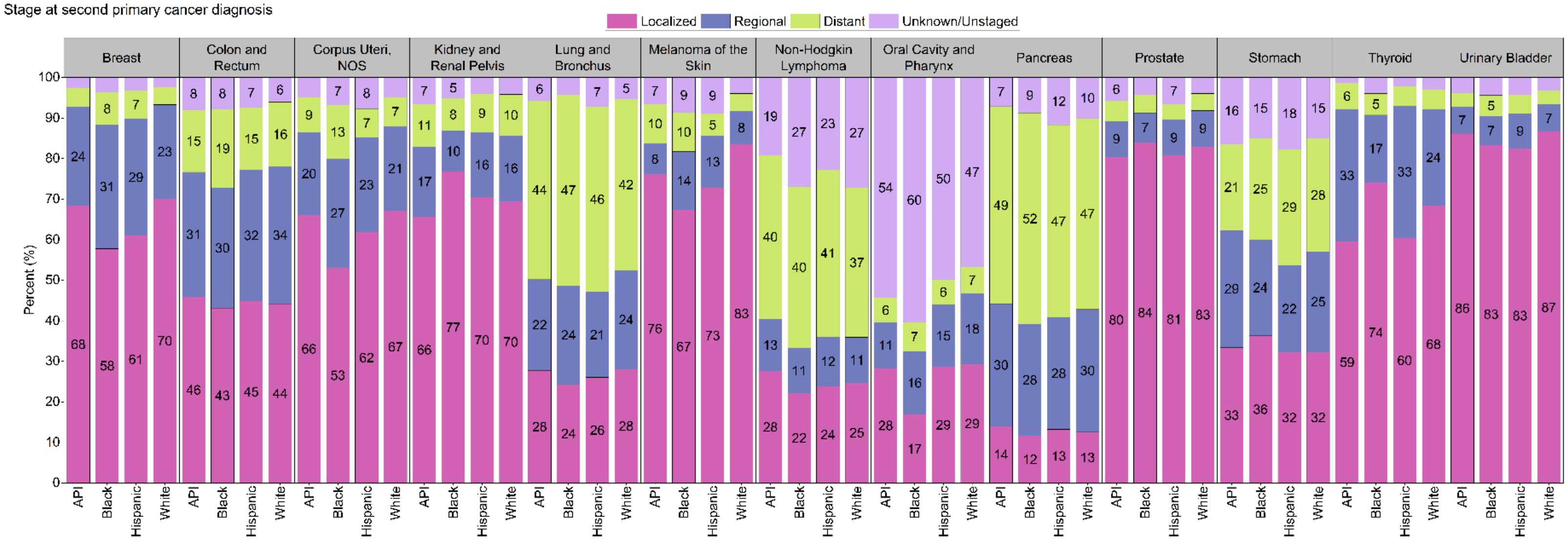

(D) Second primary cancer subtypes

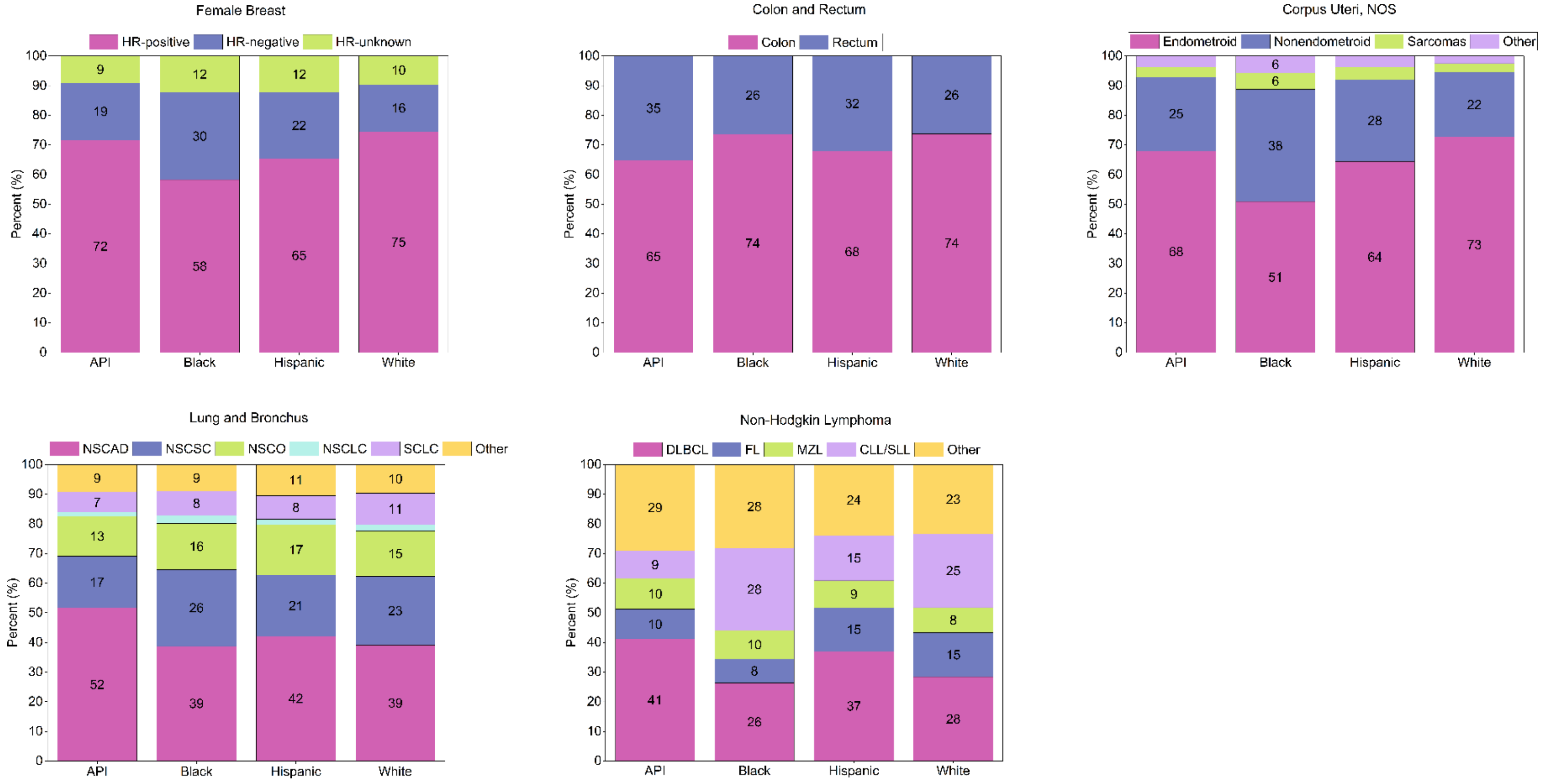

Abbreviation: API, Asian or Pacific Islander, NSCAD, Non-small cell adenocarcinoma; NSCSC, Non-small cell squamous cell carcinoma; NSCO, Non-small cell cancer, not otherwise specified; NSCLC, Large cell carcinoma; SCLC, Small cell lung cancer; DLBCL, Diffuse large B-cell lymphoma; FL, Follicular lymphoma; MZL, Marginal zone lymphoma; CLL/SLL, Chronic lymphocytic leukemia/small lymphocytic lymphoma
NOTE All race categories are exclusive of Hispanic ethnicity

© 2023 Sung H, et al. *JAMA Network Open*.

eFigure 2. Five-Year, Age-Standardized Relative Survival of Second Primary Cancers by Race and Ethnicity, Overall and by Stage

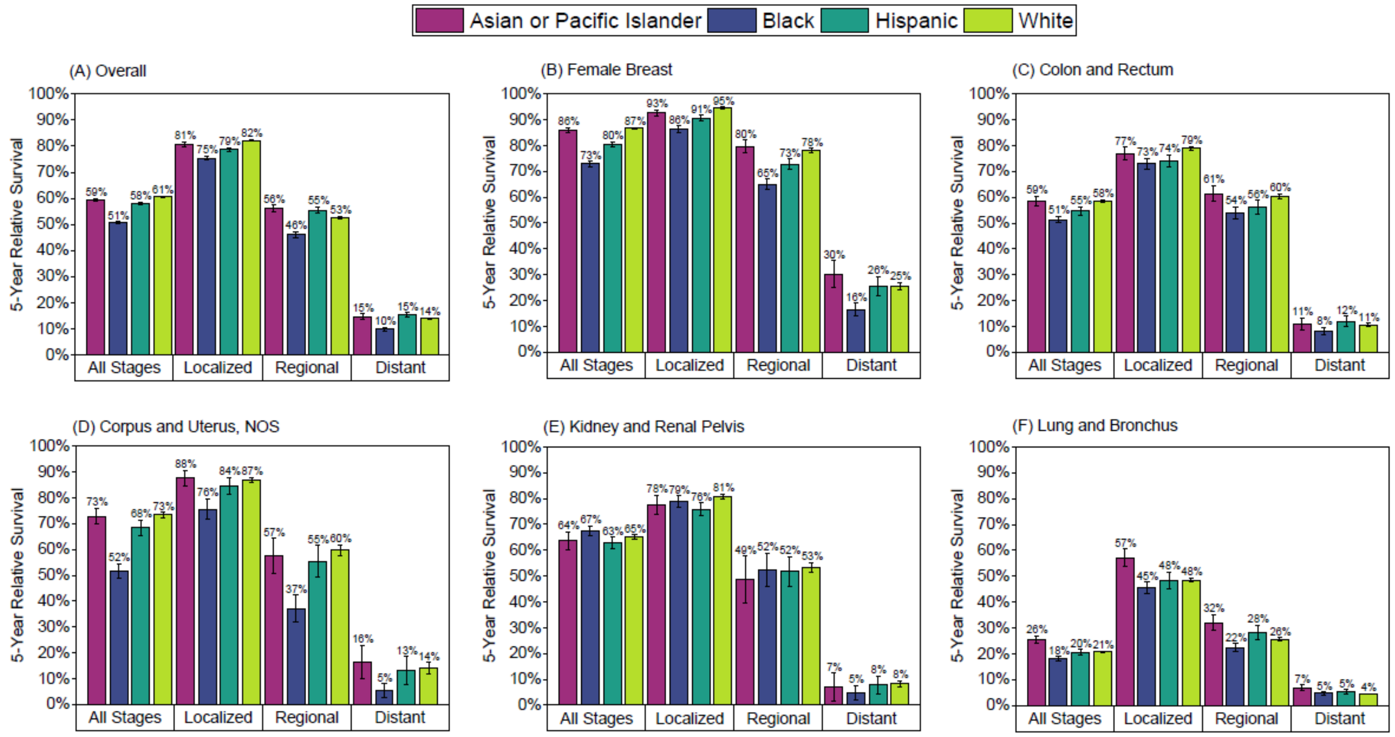

© 2023 Sung H, et al. *JAMA Network Open*.

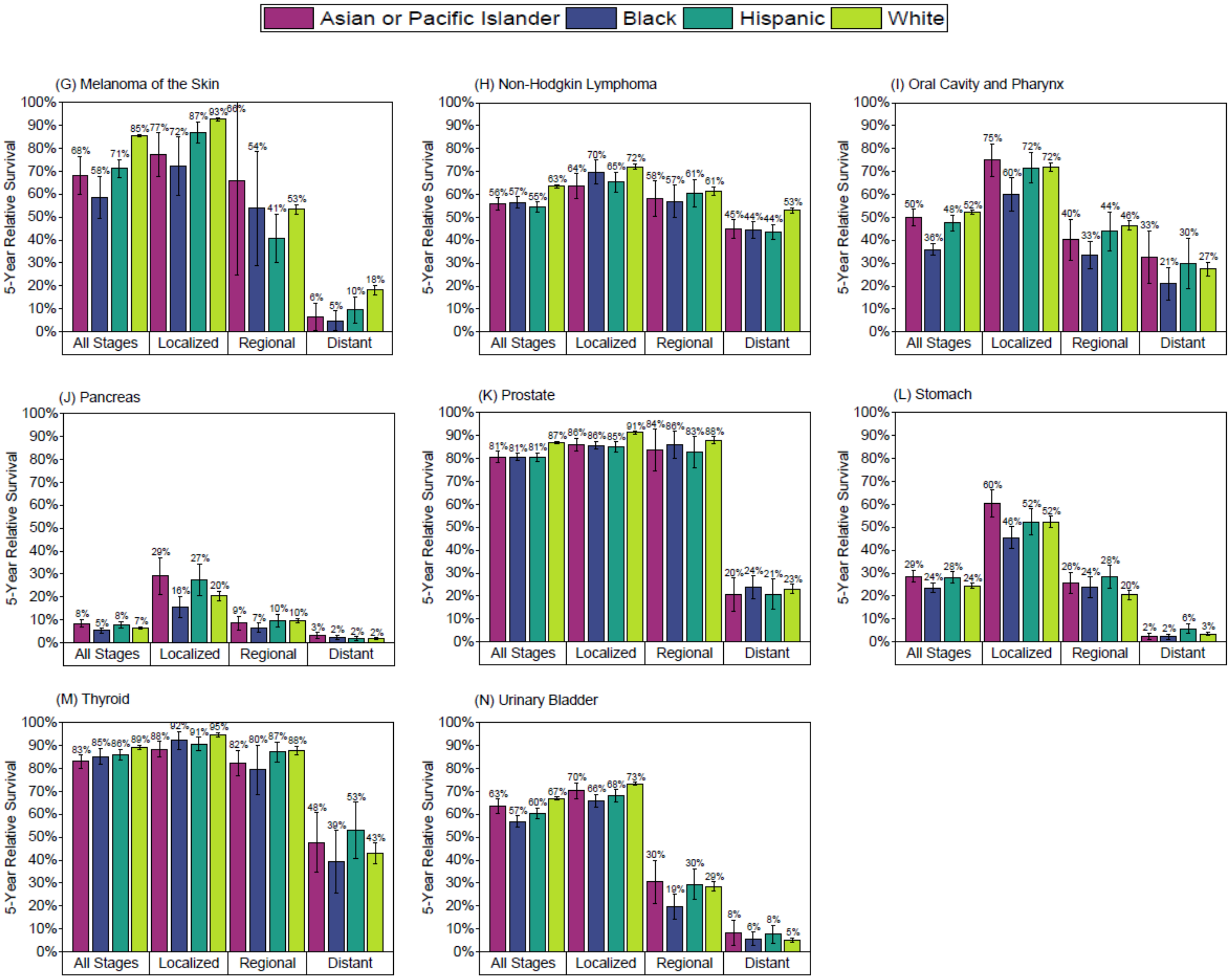

© 2023 Sung H, et al. *JAMA Network Open*.

eFigure 3. Association of Race and Ethnicity With the Risk of Cancer Death Among Persons With Second Primary Cancers (SPCs)

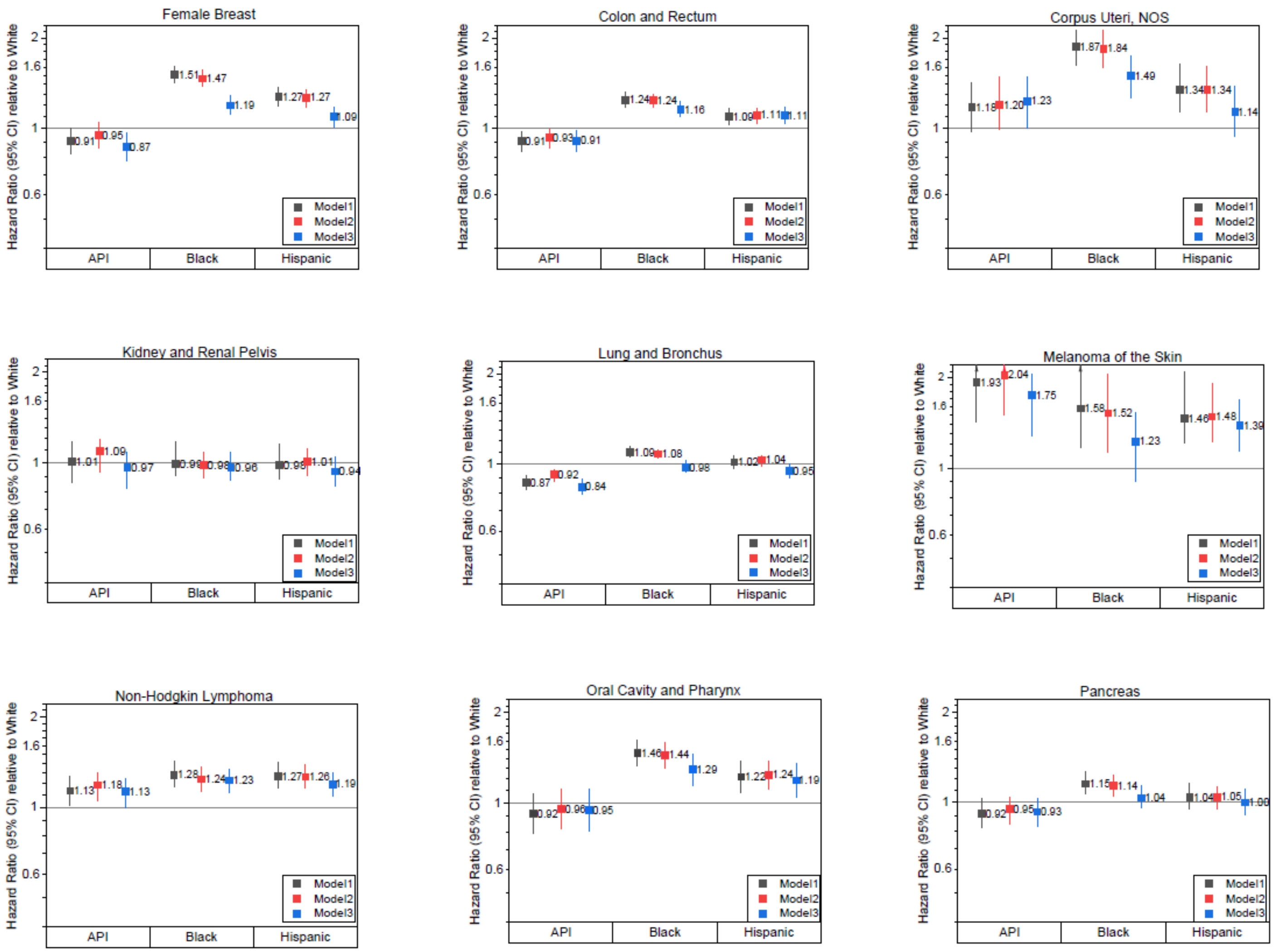

© 2023 Sung H, et al. *JAMA Network Open*.

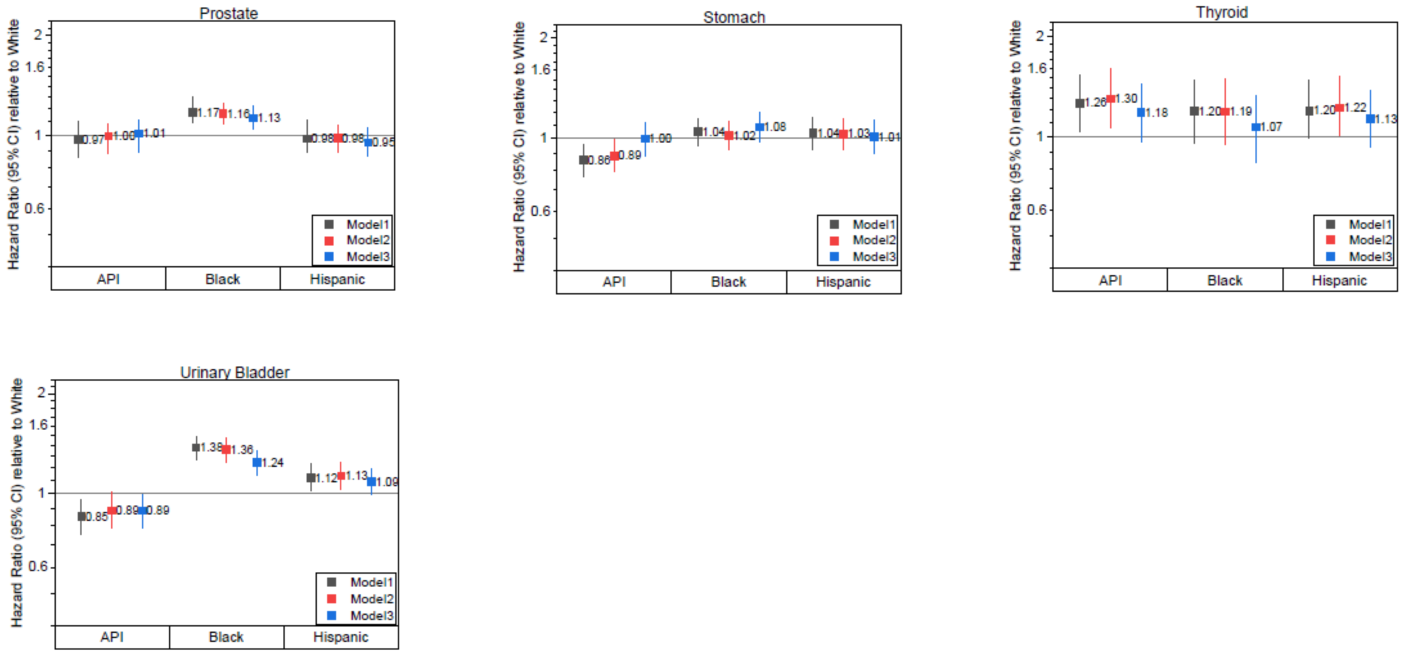

Abbreviation: API, Asian or Pacific Islander

NOTE All race categories are exclusive of Hispanic ethnicity. In Cox proportional hazards regression, sequentially adjusted covariates included sex (male, female), prior cancer type/system (eTable2 for details), prior cancer stage (localized, regional, distant, unknown/unstaged/blank), year (2000-2004, 2005-2009, 2010-2013) and age (5-year interval) at second primary cancer diagnosis for Model1; additionally county-level household income (<$35,000-$59,000, $60,000-$74,999, $75,000+, unknown), county-level urbanity (large metropolitan, small metropolitan, nonmetropolitan, unknown) for Model2; and additionally SPC stage, SPC subtype (eTable3 for details), SPC treatment including receipt of surgery, receipt of radiotherapy, and receipt of chemotherapy for SPC for Model3. All point estimates and 95% CIs along with percentage reductions in HRs between Model1 and Model3 can be found in **eTable 6.**

© 2023 Sung H, et al. *JAMA Network Open*.

eFigure 4. Association of Race and Ethnicity With the Risk of Cardiovascular Death Among Persons With Second Primary Cancers (SPCs)

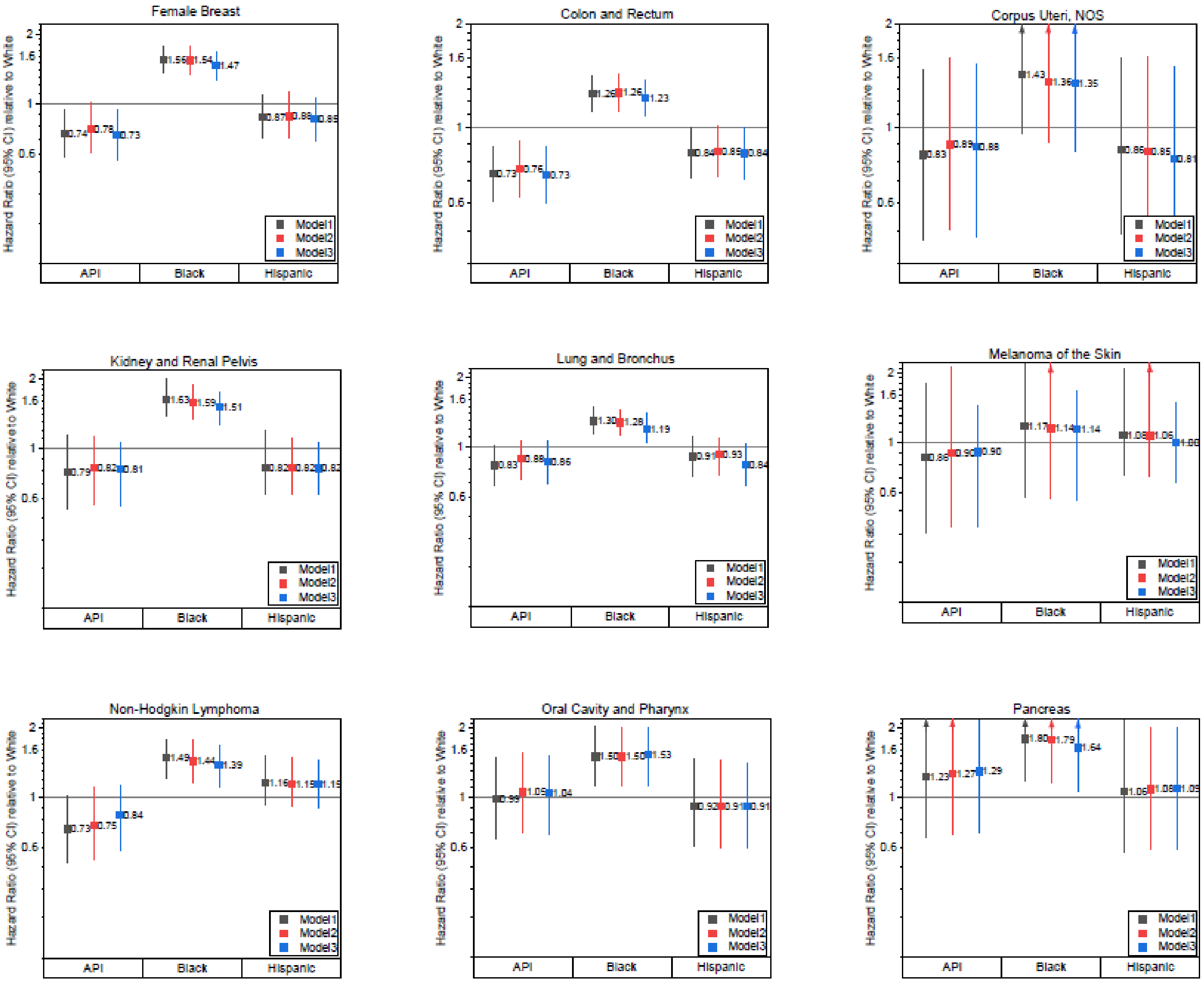

© 2023 Sung H, et al. *JAMA Network Open*.

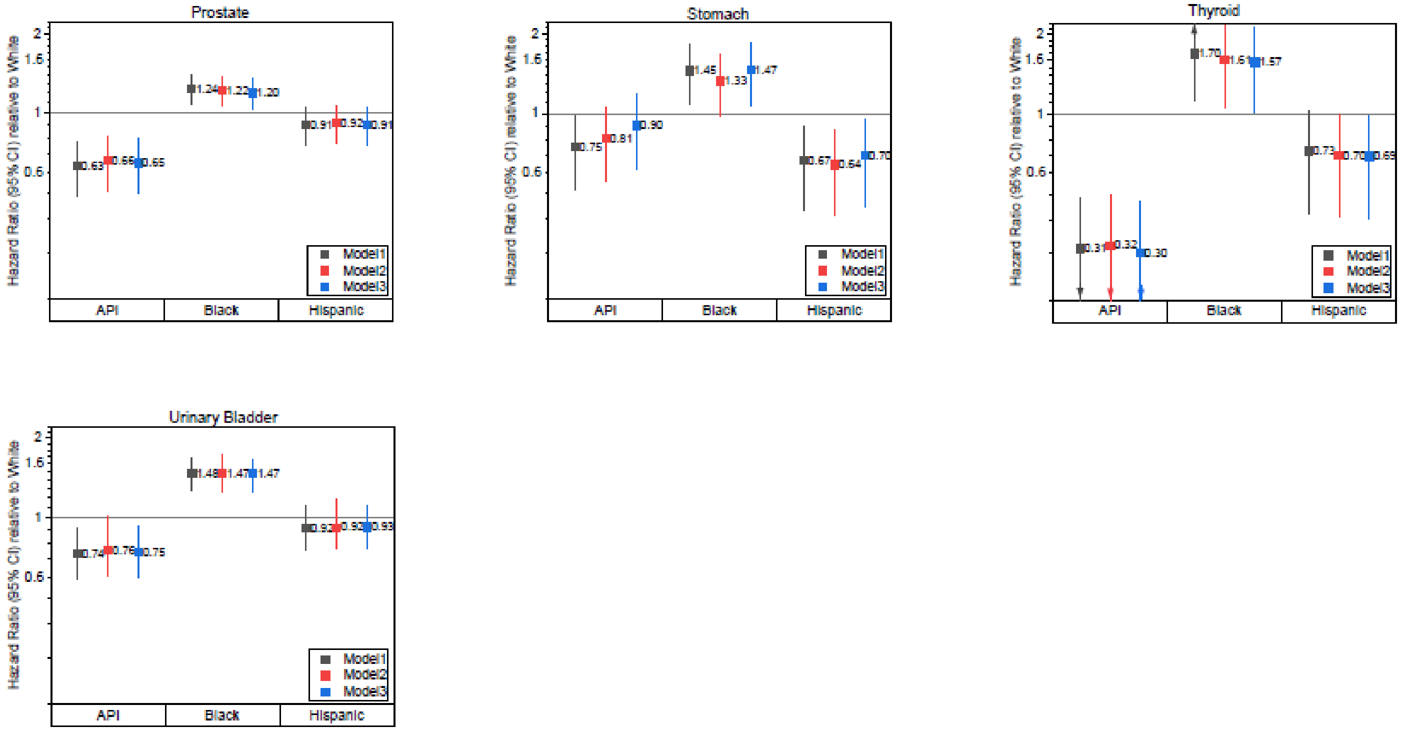

Abbreviation: API, Asian or Pacific Islander

NOTE All race categories are exclusive of Hispanic ethnicity. In Cox proportional hazards regression, sequentially adjusted covariates included sex (male, female), prior cancer type/system (eTable2 for details), prior cancer stage (localized, regional, distant, unknown/unstaged/blank), year (2000-2004, 2005-2009, 2010-2013) and age (5-year interval) at second primary cancer diagnosis for Model1; additionally county-level household income (<$35,000-$59,000, $60,000-$74,999, $75,000+, unknown), county-level urbanity (large metropolitan, small metropolitan, nonmetropolitan, unknown) for Model2; and additionally SPC stage, SPC subtype (eTable3 for details), SPC treatment including receipt of surgery, receipt of radiotherapy, and receipt of chemotherapy for SPC for Model3. All point estimates and 95% CIs along with percentage reductions in HRs between Model1 and Model3 can be found in **eTable 7.**

© 2023 Sung H, et al. *JAMA Network Open*.

eFigure 5. Associations of Race and Ethnicity[a] and the Risk of Cancer or Cardiovascular Death by Participants' Characteristics Among Persons With Second Primary Cancers (SPCs)

(A) Risk of cancer death relative to Whites

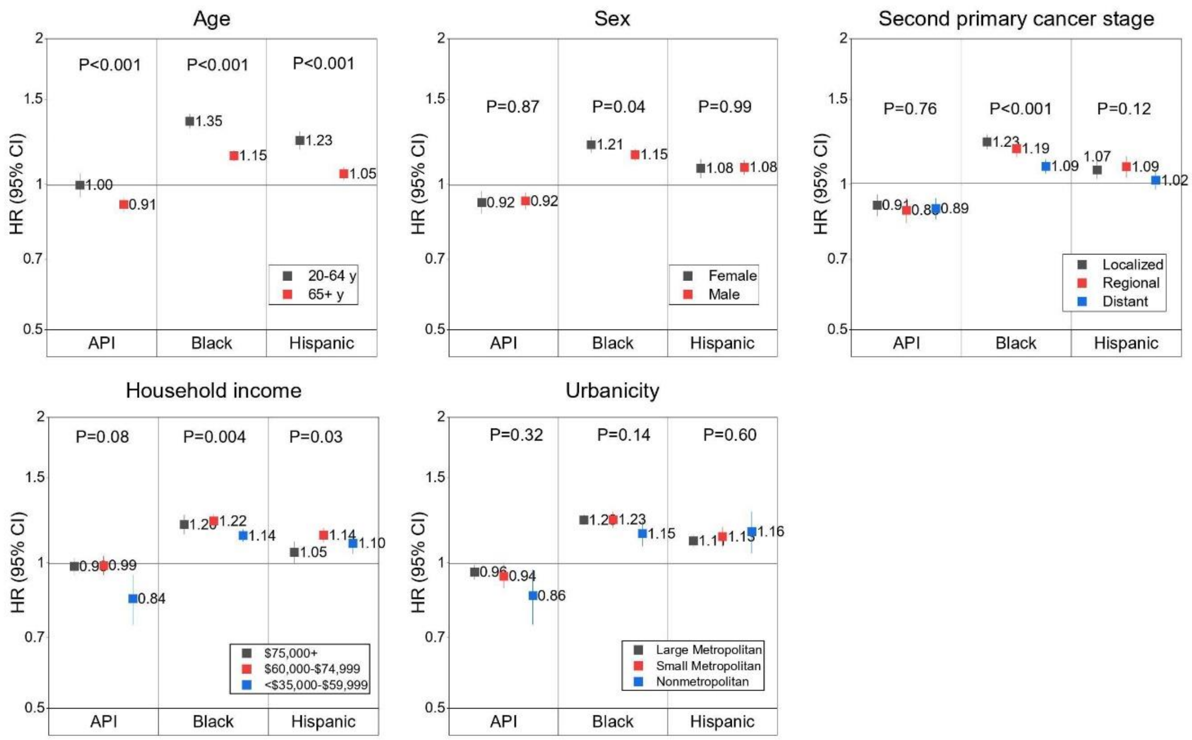

(B) Risk of cardiovascular disease death relative to Whites

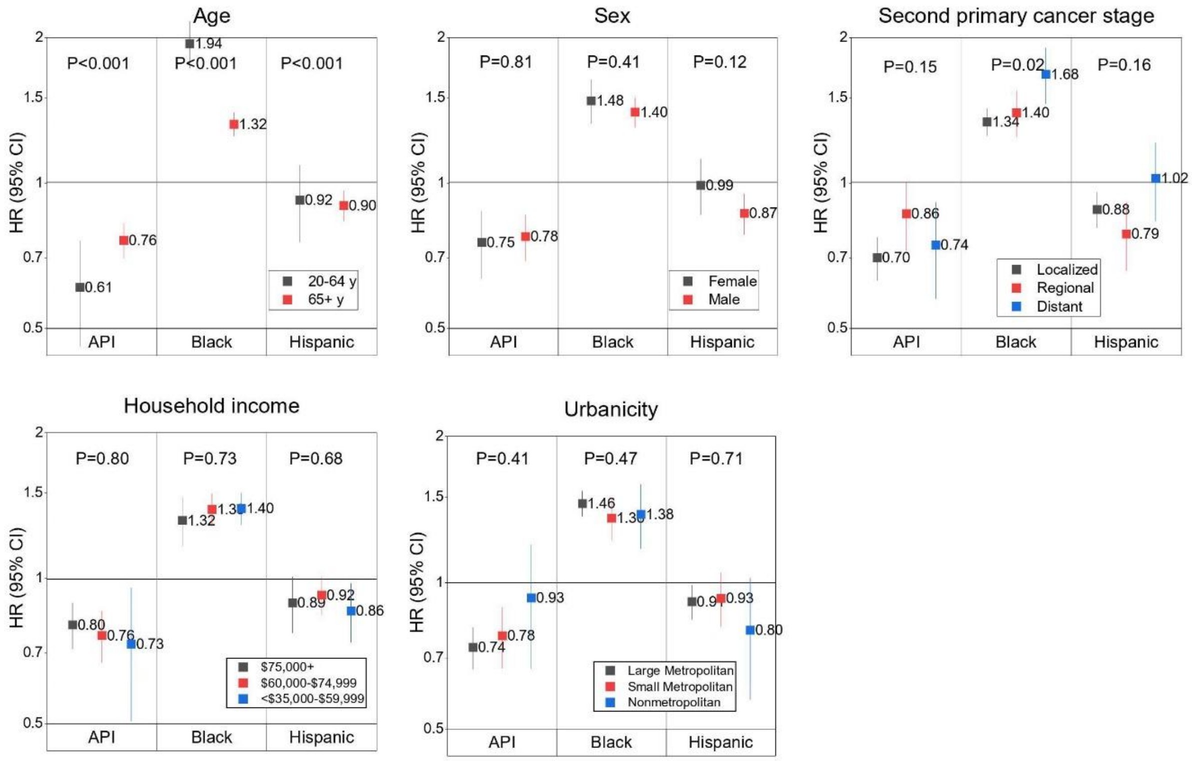

Abbreviation: API, Asian or Pacific Islander

© 2023 Sung H, et al. *JAMA Network Open*.

NOTE All race categories are exclusive of Hispanic ethnicity. Cox proportional hazard models were adjusted for sex (male, female), prior cancer type/system (**eTable 3** for details), prior cancer stage (localized, regional, distant, unknown/unstaged/blank) and year (2000-2004, 2005-2009, 2010-2013) at second primary cancer diagnosis. Interactions between race/ethnicity and the variable of interest was tested using a likelihood ratio test and the strength of the evidence for interaction was indicated with P-values.

© 2023 Sung H, et al. *JAMA Network Open*.
