# Supplementary material for: Racial and Ethnic Disparities in Survival Among People With Second Primary Cancer in the US
Source: JAMA Netw Open. 2023 Aug 4;6(8):e2327429. doi: 10.1001/jamanetworkopen.2023.27429 (PMC10403787; doi:10.1001/jamanetworkopen.2023.27429)
Supplement: Supplement 2. — Data Sharing Statement [file jamanetwopen-e2327429-s002.pdf]

Data Sharing Statement

Sung. Racial and Ethnic Disparities in Survival Among People With Second Primary Cancer in the US. *JAMA Netw Open*. Published August 04, 2023.
doi:10.1001/jamanetworkopen.2023.27429

Data
Data available: Yes
Data types: Deidentified participant data
How to access data: The database is publicly available and can be obtained upon user authentication from the Surveillance, Epidemiology, and End Results (SEER) Program at National Cancer Institute (https://seer.cancer.gov/data/).
When available: With publication

Supporting Documents
Document types: None

Additional Information
Who can access the data: The database is publicly available and can be obtained upon user authentication from the Surveillance, Epidemiology, and End Results (SEER) Program at National Cancer Institute (https://seer.cancer.gov/data/).
Types of analyses: The database is publicly available and can be obtained upon user authentication from the Surveillance, Epidemiology, and End Results (SEER) Program at National Cancer Institute (https://seer.cancer.gov/data/).
Mechanisms of data availability: The database is publicly available and can be obtained upon user authentication from the Surveillance, Epidemiology, and End Results (SEER) Program at National Cancer Institute (https://seer.cancer.gov/data/).
Any additional restrictions: The database is publicly available and can be obtained upon user authentication from the Surveillance, Epidemiology, and End Results (SEER) Program at National Cancer Institute (https://seer.cancer.gov/data/).
